# Network analysis of the metabolome and transcriptome reveals novel regulation of potato pigmentation

**DOI:** 10.1093/jxb/erv549

**Published:** 2016-01-04

**Authors:** Kyoungwon Cho, Kwang-Soo Cho, Hwang-Bae Sohn, In Jin Ha, Su-Young Hong, Hyerim Lee, Young-Mi Kim, Myung Hee Nam

**Affiliations:** ^1^Environmental Risk and Welfare Research Team, Korea Basic Science Institute (KBSI), Seoul 02855, Republic of Korea; ^2^Highland Agriculture Research Institute, National Institute of Crop Science, Rural Development Administration, Pyeongchang 25342, Republic of Korea; ^3^Omics System Research Team, Korea Basic Science Institute (KBSI), Seoul 03759, Republic of Korea; ^4^Department of Agricultural Biotechnology, National Academy of Agricultural Science, Rural Development Administration (RDA), Jeongju 54874, Republic of Korea

**Keywords:** Anthocayanin, metabolomics, colored potato, RNA-seq, ultrapressure liquid chromatography quadrupole time-of-flight mass spectrometry (UPLC-Q-TOF-MS).

## Abstract

To gain insights into the regulatory networks related to anthocyanin biosynthesis and identify key regulatory genes, we performed an integrated analysis of the transcriptome and metabolome in sprouts germinated from three colored potato cultivars: light-red Hongyoung, dark-purple Jayoung, and white Atlantic. We investigated transcriptional and metabolic changes using statistical analyses and gene–metabolite correlation networks. Transcript and metabolite profiles were generated through high-throughput RNA-sequencing data analysis and ultraperformance liquid chromatography quadrupole time-of-flight tandem mass spectrometry, respectively. The identification and quantification of changes in anthocyanin were performed using molecular formula-based mass accuracy and specific features of their MS^2^ spectra. Correlation tests of anthocyanin contents and transcriptional changes showed 823 strong correlations (correlation coefficient, *R* ^2^>0.9) between 22 compounds and 119 transcripts categorized into flavonoid metabolism, hormones, transcriptional regulation, and signaling. The connection network of anthocyanins and genes showed a regulatory system involved in the pigmentation of light-red Hongyoung and dark-purple Jayoung potatoes, suggesting that this systemic approach is powerful for investigations into novel genes that are potential targets for the breeding of new valuable potato cultivars.

## Highlight

An integrated approach of metabolomics and transcriptomics was applied to understand regulatory networks associated with biosynthesis of anthocyanins that are differentially regulated in light-red- and dark-purple-colored potato cultivars.

## Introduction

To date, more than 635 anthocyanins have been identified in numerous fruits, vegetables, and flowers (Wu and Prior, 2005; He and Giusti, 2010; He *et al.*, 2010). The anthocyanin derivatives of delphinidin, petunidin, and malvidin are sources of purple and dark colors, whereas the derivatives of cyanidin and pelargonidin are the main pigments in bright-red-colored fruits (Jaakola, 2013). Although several genes encoding proteins implicated in anthocyanin biosynthesis and regulation have been identified, insights into the regulation of each anthocyanin biosynthesis pathway have remained future objectives. Recently, the integration of large-scale datasets derived from high-throughput functional genomics techniques have been applied successfully to studies on the functions of genes regulating tissue development (Persson *et al.*, 2005), environmental responses (Wang *et al.*, 2006; Cho *et al.*, 2008), and plant metabolism (Hirai *et al.*, 2007; Gutiérrez *et al.*, 2008). In particular, transcript and metabolite datasets have been combined through correlation and clustering analyses and further represented as connection networks between genes and metabolites in many plants (Urbanczyk‐Wochniak *et al.*, 2003; Hoefgen and Nikiforova, 2008; Saito *et al.*, 2008), including Arabidopsis (Hirai *et al.*, 2004, 2007), tomatoes (Alba *et al.*, 2005; Mounet *et al.*, 2009), and potatoes (Stushnoff *et al.*, 2010). The acquisition and integration of an “omics” dataset (i.e. transcriptome, proteome, and metabolome) represents a useful approach for the establishment of a strategy to identify potential genes regulating the determination of pigmentation in potatoes.

Colored potatoes have attracted research interest due to their anthocyanin content with enhanced antioxidant capacities (Truong *et al.*, 2009; Stushnoff *et al.*, 2010; Chong *et al.*, 2013). *Solanum tuberosum* cvs Hongyoung and Jayoung are pigmented potato cultivars originated from a cross made between the white-colored Atlantic and deep dark-purple-colored AG34314 cultivars through the potato breeding program of the National Institute of Highland Agriculture Research Center in 2003 (Park et al., 2009a, b). Hongyoung has a light-red skin and light-red flesh, while Jayoung has dark-purple skin and dark-purple flesh (see Supplementary Fig. S1 available at *JXB* online).

Most anthocyanins have been identified by the combined methods of ultraviolet/visible (UV/Vis) spectrometry and mass spectrometry (MS). In positive ionization mode, the [M^+^] ion and mass fragmentation patterns of anthocyanins are the same as the [M+H]^+^ ion and fragmentation patterns of flavonol (Wu and Prior, 2005; Sun *et al.*, 2012). Anthocyanins (480–540nm) and non-anthocyanin phenolic compounds (<400nm) have maximum absorbance at different UV/Vis wavelengths. Thus, a liquid chromatography (LC) mass spectrometer equipped with a photodiode array detector is usually used to distinguish anthocyanin and flavonol glycosides (Lin *et al.*, 2011; Sun *et al.*, 2012). A recent study showed that MS data acquired in the negative ionization mode using ultrahigh-performance LC with high-resolution MS provided a series of characteristic ions for anthocyanins (e.g. [M-2H]^–^, [M-2H+H_2_O]^–^, and formic acid adducts) (Sun *et al.*, 2012), suggesting that the integrative analysis of mass ions and mass fragmentations acquired from both the positive and negative modes can distinguish and identify anthocyanin and flavonol glycosides.

In this study, we explored the regulatory networks of anthocyanin biosynthesis in colored potatoes at the level of the transcriptome and metabolome. We focused on the differential expression of anthocyanin metabolites and their regulatory genes in light-red Hongyoung and dark-purple Jayoung potatoes compared with those of a white Atlantic potato cultivar. Connection networks were mapped on the basis of correlation analyses between metabolites and transcripts to highlight the regulatory genes associated with anthocyanin metabolites. Our findings provide new insights into the molecular mechanisms associated with the biosynthesis and regulation of anthocyanin in the pigmentation of potatoes, and highlight the usefulness of an integrated approach for understanding this process.

## Materials and methods

### Plant material

Medium-sized (80–150g) potato tubers from three different potato cultivars, Hongyoung, Jayoung, and Atlantic, were stored at a low temperature (4 °C) for 4 months after harvesting in the Dae-Gwal-Lyeong area (800 m above sea level), Korea. After storage, sprouts of potato tubers were induced at room temperature for 1 month with scattered light conditions. Whole sprouts were collected, immediately frozen in liquid nitrogen, and then stored at –80 °C prior to metabolite extraction.

### Metabolite extraction

Frozen spouts of potatoes (200mg) were ground using a bead beater (4.5ms^−1^, 25s, three repetitions, MP 24X4, FastPrep-24; MP Biochemicals), suspended in methanol (600 μl) with a 0.125% formic acid solution, kept at 4 °C for 30min, sonicated at 4 °C for 20s (20kHz, 250W, three repetitions, Bioruptor-KRB-01; Bop-Medical Science), and centrifuged at 3000rpm for 15min at 4 °C. Finally, the supernatant solution was centrifuged at 13 000rpm for 10min at 4 °C and used for metabolomics analysis.

### Metabolite profiling using ultraperformance LC quadrupole time-of-flight tandem MS (UPLC-Q-TOF-MS)

Metabolite profiling was conducted using a UPLC system (ACQUITY UPLC; Waters, Milford, MA, USA) and hybrid Q-TOF tandem mass spectrometry (Triple-TOF-MS) (Triple TOF 5600 system; AB SCIEX, Concord, ON, Canada). Chromatographic separation was performed on an ACQUITY UPLC BEH C18 column (2.1 mm×100 mm×1.7 μm; Waters) using mobile phase A (0.1% formic acid in deionized water) and mobile phase B (0.1% formic acid in acetonitrile). Mobile phase B was increased linearly from 3% at 0min to 50% at 3min to 70% at 4min to 100% at 10min, and then held at 100% until 10.5min. Finally, solvent B was decreased to 3% at 11min and held at 3% until 12min. The flow rate was maintained at 0.4ml min^–1^. Mass data acquisition was performed in both positive [electrospray ionization-positive (ESI^+^)] and negative (ESI^–^) modes using the following parameters: ion spray voltage of 5.5kV in ESI^+^ and –4.5kV in ESI^–^; nebulizer gas (gas 1) of 55 psi; heater gas (gas 2) of 65 psi; curtain gas of 30 psi; turbo spray temperature of 600 °C; and declustering potential of 100V in ESI^+^ and –100V in ESI^–^. For TOF MS^2^ data, information-dependent acquisition was used with the following conditions: survey scans of 250ms; product ion scans of 70ms; high-resolution mode; declustering potential of 90V; collision energy of 35V in ESI^+^ (–35V in ESI^–^); and collision energy spread of 15V. The TOF-MS and information-dependent acquisition scan was operated with the mass range of *m*/*z* 50–1600. TOF-MS and product ion calibration was performed in both high-sensitivity and high-resolution modes using a calibrant delivery system prior to analysis.

LC-MS data files (Wiff format files) including MS and MS^2^ spectra data were converted to mzXML files using MSConvert in the Proteowizard software (version 3.04999) (Patti *et al.*, 2012). The converted raw data were further processed using MZmine software (version 2.10) and outputted as a retention time *m*/*z* dataset.

### Multivariate statistical analysis

The intensities of mass peaks for each sample were sum-normalized and Pareto-scaled using the SIMCA-P^+^ software package (version 12.0; Umetrics, Umeå, Sweden). Principal component analysis (PCA) and orthogonal partial least-squares discriminant analysis (OPLS-DA) with data from 18 samples (three cultivars×six biological replicates) were performed to observe differences in metabolic composition among the three potato cultivars. The reliability correlation [*p*(corr)] values of all metabolites from the S-plot of the OPLS-DA were extracted using the first component. We selected metabolites satisfying the following criteria as potential markers: (i) high confidence [|*p*(corr)|>0.6] in discriminations between Hongyoung and Atlantic, between Jayoung and Atlantic, and between Jayoung and Hongyoung; (ii) mean intensities in one potato cultivar significantly different from those of another cultivar (*P*<0.05); and (iii) a minimum of a 2-fold change. The *P* value was calculated using an independent two-sample *t*-test.

### Targeted selection of anthocyanins and their intermediates

Targeted selection of anthocyanins and their intermediates was performed based on follow information: (i) molecular formula and exact mass information of the compounds on phenylprophanoid and flavonoid pathways were established throughout the KEGG (http://www.genome.jp/kegg) and PMN (plant metabolic network, http://www.plantcyc.org) databases and literary references (see Supplementary Fig. S2 and Table S2 available at *JXB* online) (He *et al.*, 2010; Stushnoff *et al.*, 2010; Jaakola, 2013); (ii) specific daughter ions of anthocyanins through literary references on similar compounds (Sun *et al.*, 2012); and (iii) MS^2^ spectra of standard compounds and metabolome databases, including METLIN (http://metlin.scripps.edu/) and LIPD MAPS (http://www.lipidmaps.org/). Finally, fragmented or adducted mass features were analyzed for selected ions. The identification of anthocyanin-related compounds is summarized in Supplementary Table S3 available at *JXB* online.

### RNA sequencing (RNA-seq) data analysis

RNA-seq paired-end libraries were prepared using the Illumina TruSeq RNA Sample Preparation Kit version 2 (Illumina, San Diego, CA, USA). Starting with total RNA isolated using PureLink^®^ Plant RNA Reagent (Life Technologies Korea LLC, Seoul, Korea), mRNA was purified using poly(A) selection or rRNA depletion; next, RNA was chemically fragmented and converted into single-stranded cDNA using random hexamer priming. Next, the second strand was generated to create double-stranded cDNA. Library construction began with the generation of blunt-end cDNA fragments from double-stranded cDNA. Then, an A base was added to the blunt ends to make them ready for the ligation of sequencing adapters. After size selection of the ligation products, the ligated cDNA fragments that contained adapter sequences were enhanced via PCR using adapter-specific primers. The library was quantified with a KAPA library quantification kit (Kapa biosystems KK4854) following the manufacturer’s instructions. Each library was loaded onto the Illumina Hiseq2000 platform and high-throughput sequencing was performed to ensure that each sample met the desired average sequencing depth. Image analysis and base calling were performed using the Illumina pipeline with default settings.

For mRNA sequencing, total RNA (10 μg) was isolated from sprouts of Atlantic, Hongyoung, and Jayoung using a PureLink^®^ RNA Mini kit (Life Technologies Korea LLC) and used to create normalized cDNA and PCR-amplified datasets according to the Illumina RNA-seq protocol; then, the RNA was sequenced by Illumina HiSeq2000 (242M 100bp paired-end reads). Sequence data with base-pair qualities in the upper Q ≥20 were extracted by SolexaQA. Trimming resulted in reads with a mean length of 80.14bp across all samples; a minimum length of 25bp was applied during sequence trimming. The gene annotation used *S. tuberosum* Group Phureja DM1-3 516R44 (CIP801092) Genome Annotation version 3.4 mapped to the pseudomolecule sequence (PGSC_DM_v3_2.1.10_pseudomolecules.fa) downloaded from Solanaceae Genomics Resource at Michigan State University (http://solanaceae.plantbiology.msu.edu/pgsc_download.shtml) (Consortium, 2011)

### Transcript profiles and annotation

mRNA libraries generated from each sample were sequenced using Illumina HiSeq2000 (100bp paired ends). Reads for each sequence tag were mapped to the reference with the Bowtie software (Langmead *et al.*, 2009). The number of mapped clean reads for each gene was counted and normalized using the DESeq package in R (Anders and Huber, 2010). Only the genes that mapped with read counts of 100 or above in all experimental samples were retained for further analysis. Fold change and binomial tests were used to identify differentially expressed genes between each sample. The false discovery rate calculated via DESeq was applied to identify the threshold of the *P* value in binomial tests and analyses.

Gene Ontology (GO) and KEGG pathway functional enrichment analyses were performed via the Gene Ontology Database and DAVID (http://david.abcc.ncifcrf.gov/tools.jsp), respectively (Huang *et al.*, 2008). GO consists of terms that provide a more global representation of gene functions using a controlled vocabulary; DAVID comprises web-accessible programs that provide a comprehensive set of functional annotation tools that can be used by investigators to understand the biological meaning behind a large list of genes. The gene lists generated by annotated TAIR (The Arabidopsis Information Resource) ID of transcripts of up- and down-regulated differentially expressed genes were classified into MapMan BINs using the MapCave tool (http://mapman.gabipd.org/web/guest/mapcave), which is linked with three databases (*Arabidopsis thaliana* TAIR8, *Arabidopsis thaliana* TAIR9, and TAIR release 10) (see Supplementary Table S4 available at *JXB* online).

### Integrative analysis of metabolome and transcriptome

Pearson correlation coefficients were calculated for metabolome and transcriptome data integration. For this, the mean of all biological replicates of each cultivar in the metabolome data and the mean value of expression of each transcript in the transcriptome data were calculated. The fold changes in each pigmented potato (Hongyoung and Jayoung) were then calculated in both the metabolome and transcriptome data and compared with the control cultivar (Atlantic). Finally, the coefficients were calculated from log_2_(fold change) of each metabolite and log_2_(fold change) of each transcript using the EXCEL program (see Supplementary Table S5 available at *JXB* online). Correlations corresponding to a coefficient with *R* ^2^>0.9 were selected (see Supplementary Table S6 available at *JXB* online). Metabolome and transcriptome relationships were visualized using Cytoscape (version 2.8.2).

## Results

### Metabolic differences among the three potato cultivars

**Fig. 1.**
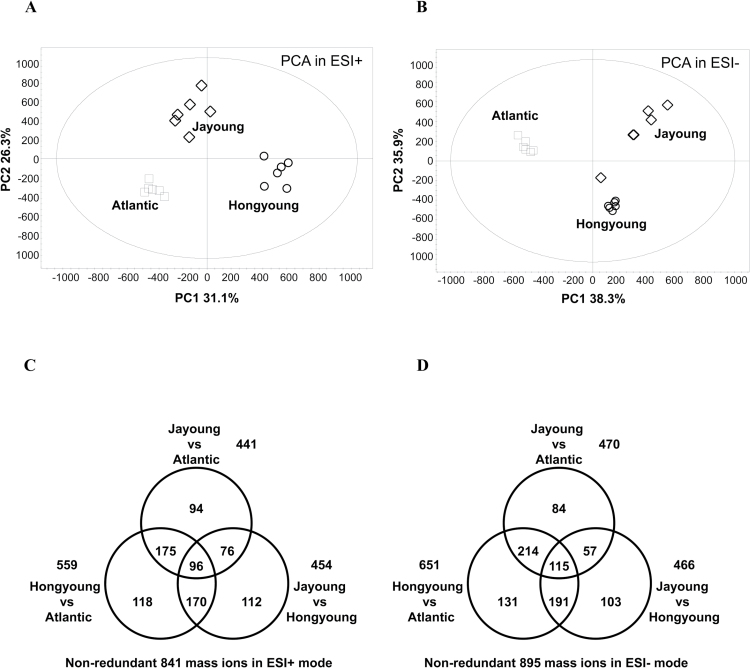
PCA score plot of colored potatoes and numbers of potential markers for each. PCA score plots were derived from metabolite ions acquired from ESI^+^ (A) and ESI^–^(B) mode. Potential markers were selected by comparing quantitative differences of mass ions in ESI^+^ (C) and ESI^–^ (D) mode between Hongyoung and Atlantic, between Jayoung and Atlantic, and between Hongyoung and Jayoung.

To compare the metabolite composition involved in the pigmentation of the three different potato cultivars, datasets obtained from UPLC-Triple-TOF-MS in the ESI^+^ (ESI^–^) mode were subjected to PCA. The results showed that the three potato cultivars were clearly separated in the PC1×PC2 score plots (Fig. 1A, B). Indeed, the first principal component (PC1) in ESI^+^ mode (31.1% of the total variables) and PC1 and PC2 in ESI^–^ (38.3 and 35.9%, respectively) were clearly separated between Hongyoung and Atlantic. The differences between Jayoung and Atlantic resulted from PC2 (26.3% variables) in ESI^+^ mode and PC1 (38.3%) in ESI^–^ mode. Furthermore, score plots and S-plots of OPLS-DA were used for modeling the differences between two potato cultivars (see Supplementary Fig. S3 available at *JXB* online). The selection of variables responsible for the differences was performed through statistical analysis as described in Materials and methods. A total of 556 (651), 441 (470), and 454 (466) mass ions were selected between Hongyoung and Atlantic, between Jayoung and Atlantic, and between Jayoung and Hongyoung in the ESI^+^ (ESI^–^) mode, respectively. In total, 841 and 895 mass ions were selected in the ESI^+^ and ESI^–^ modes, respectively (Fig. 1C, D).

### Differential accumulation of anthocyanin derivatives between Hongyoung and Jayoung

Anthocyanins are glycosides and acylglycosides of anthocyanidin aglycones that are biosynthesized through the flavonoid pathway via the phenylpropanoid pathway (Stushnoff *et al.*, 2010; Jaakola, 2013). Cyanidin (Cy), delphinidin (Dp), pelargonidin (Pg), peonidin (Pn), petunidin (Pt), and malvidin (Mv) are six common anthocyanins that are grouped according to the hydroxyl pattern or methoxy substitutions of B ring (Supplementary Fig. S2). Among them, the anthocyanin derivatives Dp, Pn, and Mv are sources of purple and dark colors, whereas the derivatives of Cy and Pg are the main pigments in bright-red-colored fruits (Jaakola, 2013).

**Fig. 2.**
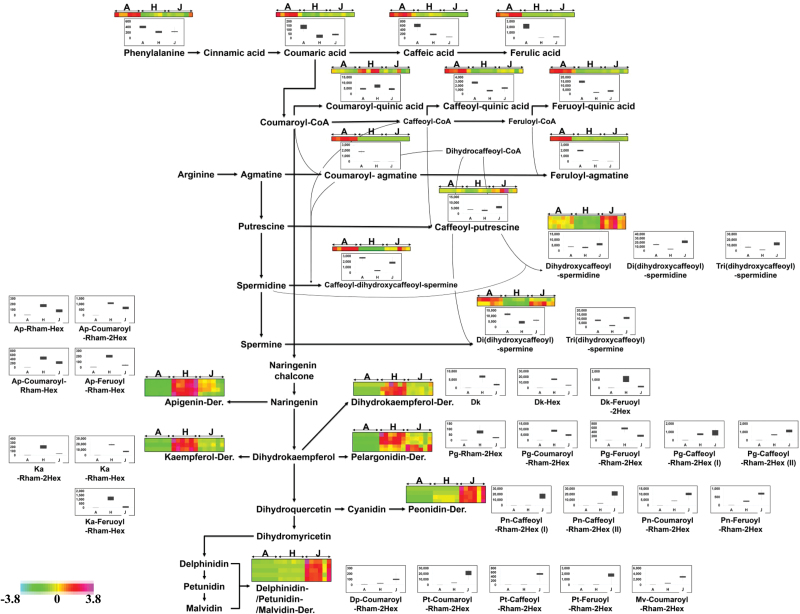
Biosynthetic pathway of anthocyanins. This pathway is constructed based on the KEGG pathway and literary references. Dk, dihydrokaempferol; Ka, kaempferol; Ap, apigenin; Pg, pelargonidin; Pt, petunidin; Pn, peonidin; Dp, delphinidin; Mv, malvidin; Rham, rhamnose; Hex, hexose; Coumaroyl, coumaric acid; Caffeoyl, caffeic acid; Feruoyl, ferulic acid; A, Atlanic potato; H, Hongyoung potato; J, Jayoung potato. Each colored cell represents the normalized intensity of each compound ion according to the color scale (six biological replicates×three cultivars, *n*=18). Box-and-whisker plots are shown for changes of phenylprophanoids, polyamine conjugates, flavone glycosides, flavonol glycosides, dihydroflavonol glycosides, and anthocyanins in each potato cultivar. Maximum and minimum values of a metabolite among six biological replicates are represented at the upper and lower ends of the whisker, respectively, and their 75th and 25th percentiles are represented at upper and lower ends of the box, respectively.

Among the mass ion peaks detected in our metabolomics analysis, anthocyanins and their intermediates were selected using product-ion scanning and precursor-ion scanning based on their molecular formula in MS^1^ and MS^2^ spectral data, respectively (see Supplementary Fig. S4 available at *JXB* online). The selected peaks were identified by interpretation of their MS^2^ fragment patterns and are summarized in Supplementary Table S3. The identified anthocyanins and their relevant compounds were rearranged to their corresponding positions in an anthocyanin biosynthesis pathway established based on KEGG, PMN, and literature references. Figure 2 shows that the composition of compounds on the anthocyanin biosynthesis pathways were specifically different depending on the potato cultivars (i.e. white Atlantic, light-red Hongyoung, and dark-purple Jayoung). In particular, the compositions of flavonoids downstream of the phenylpropanoid pathway were highly different between Hongyoung and Jayoung, indicating that these metabolites might play a crucial role in determining the pigmentation in potato. Apigenin (Ap), kaempferol (Ka), dihydrokaempferol (Dk), and Pg derivatives were shown to be most abundant in Hongyoung, whereas Pn, Pt, Dp, and Mv derivatives were shown to be the more abundant in Jayoung.

### Differential expression of genes among Atlantic, Hongyoung, and Jayoung

Transcriptome analysis of sprouts showed that 756 and 519 transcripts had at least a 4-fold change in Hongyoung and Jayoung, respectively, compared with Atlantic. There were 482 and 274 up-regulated and down-regulated transcripts in Hongyoung, and 248 and 273 in Jayoung, respectively (Supplementary Fig. S5 available at *JXB* online). In total, 1044 non-redundant transcripts were collected and classified into 20 groups (main BINs) based on their annotated functions (Table 1). The transcripts were categorized as unknown (37.7%), regulation of protein activity (10.3%), stress (9.6%), other metabolism (7.3%), transcriptional regulation (5.7%), secondary metabolism (5.3%), and signaling (4.0%) (Table 1).

**Table 1.** Functional categories of the genes differentially expressed in Hongyoung and Jayoung compared with those of the corresponding control, Atlantic

BIN Code^*a*^	Annotation	Hongyoung				Jayoung				Total number of non-redundant genes	
		Up		Down		Up		Down			
		Number	%	Number	%	Number	%	Number	%	Number	%
BIN 1	Photosynthesis	1	0.2	5	1.8	1	0.4	1	0.4	6	0.6
BIN 10	Cell wall	13	2.7	8	2.9	4	1.6	2	0.7	23	2.2
BIN 11	Lipid metabolism	7	1.5	7	2.6	4	1.6	3	1.1	17	1.6
BIN 13	Amino acid metabolism	4	0.8	0	0.0	1	0.4	2	0.7	6	0.6
BIN 16	Secondary metabolism	29	6.0	16	5.8	22	8.9	7	2.6	55 (35^*b*^)	5.3
BIN 17	Hormone	16	3.3	6	2.2	5	2.0	6	2.2	30^*c*^	2.9
BIN 20	Stress	45	9.3	21	7.7	24	9.7	37	13.6	100	9.6
BIN 21	Redox	1	0.2	1	0.4	1	0.4	0	0.0	3	0.3
BIN 27.A^*d*^	RNA processing/transcription/ RNA binding	5	1.0	5	1.8	1	0.4	6	2.2	16	1.5
BIN 27.3	Regulation of transcription	34	7.1	9	3.3	14	5.7	13	4.8	60^*c*^	5.7
BIN 28	Chromatin structure/DNA synthesis and repair	6	1.2	2	0.7	5	2.0	2	0.7	12	1.1
BIN 29	Protein synthesis/folding	4	0.8	2	0.7	4	1.6	3	1.1	11	1.1
BIN 30	Signaling	15	3.1	14	5.1	11	4.5	11	4.0	42^*c*^	4.0
BIN 31	Cell	5	1.0	4	1.5	2	0.8	3	1.1	12	1.1
BIN 33	Development	12	2.5	4	1.5	4	1.6	2	0.7	18	1.7
BIN 34	Transport	23	4.8	7	2.6	4	1.6	6	2.2	36	3.4
BIN 35	Not assigned	156	32.4	118	43.1	85	34.4	125	46.0	394	37.7
BIN A^*e*^	Carbohydrate metabolism	10	2.1	4	1.5	5	2.0	3	1.1	19	1.8
BIN B^*f*^	Other metabolism	52	10.8	28	10.2	28	11.3	24	8.8	108	10.3
BIN C^*g*^	Regulation of protein activity	44	9.1	13	4.7	22	8.9	16	5.9	76	7.3
Total		482	100.0	274	100.0	247	100.0	272	100.0	1044	100.0

^*a*^ BIN codes of genes were produced according to MapMan classification using the MapCave tool (http://mapman.gabipd.org/web/guest/mapcave).

^*b*^ Represents the number of genes for anthocyanin biosynthesis.

^*c*^ Represents the number of genes that were selected to analyze the correlation test between gene and metabolite.

^*d*^ BIN 27.A includes RNA processing (BIN 27.1), transcription (27.2), and RNA binding (27.4).

^*e*^ BIN A is carbohydrate metabolism-related BINs: major carbohydrates (BIN 2), minor carbohydrates (BIN 3), glycolysis (BIN 4), fermentation (BIN 5), gluconeogenesis/glyoxylate cycle (BIN 6), OPP cycle (BIN 7), TCA/organic acid transformation (BIN 8), and mitochondrial electron transport/ATP synthesis (BIN 9).

^*f*^ BIN B is other metabolism-related BINs: nitrogen assimilation (BIN 12), S-assimilation (BIN 14), metal handling (BIN 15), cofactor/vitamin synthesis (BIN 18), tetrapyrole synthesis (BIN 19), nucleotide metabolism (BIN 23), biodegradation of xenobiotics (BIN 24), C1-metabolism (BIN 25), and miscellaneous (BIN 26).

^*g*^ BIN C is protein activity regulation-related BINs including protein targeting (BIN 29.3), protein post-translational modification (BIN 29.4), and protein degradation (BIN 29.5).

### Correlation analysis between transcripts and anthocyanin derivatives reveals the differential regulatory network of anthocyanin biosynthesis in Hongyoung and Jayoung

**Fig. 3.**
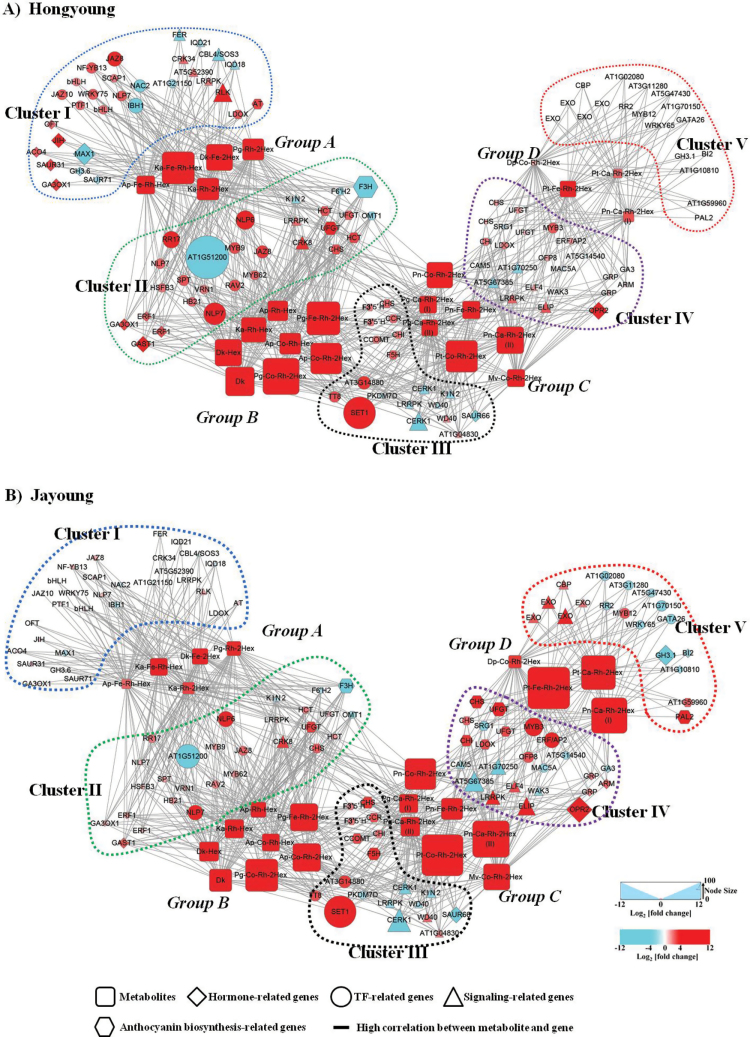
Connection network between regulatory genes and anthocyanin-related metabolites. The networks in Hongyoung (A) and Jayoung (B) were visualized with the Cytoscape software (version 2.8.2). Dk, dihydrokaempferol; Ka, kaempferol; Ap, apigenin; Pg, pelargonidin; Pt, petunidin; Pn, peonidin; Dp, delphinidin; Mv, malvidin; Rh, rhamnose; Hex, hexose; Co, coumaric acid; Ca, caffeic acid; Fe, ferulic acid.

To understand the regulatory network of anthocyanins implicated in the differential distribution of anthocyanin derivatives between Hongyoung and Jayoung, we carried out correlation tests between quantitative changes of metabolites and transcripts in the three different colored potatoes. For this, derivatives of Ka, Dk, Dp, Pg, Pn, Pt, and Mv detected in this study and transcripts categorized into flavonoid metabolism, hormone metabolism, regulation of transcription, and cell signaling were selected from the 1044 genes (Supplementary Fig. S5) differentially expressed in the three potato cultivars. In total, 22 anthocyanin derivatives and 167 transcripts (flavonoid metabolism: 35, hormone metabolism: 30, regulation of transcription: 60, and cell signaling: 42 transcripts) were subjected to Pearson correlation analysis (Supplementary Table S5). The result showed that 119 transcripts had strong correlation coefficient values (*R* ^2^>0.9) with 22 metabolites (Supplementary Table S6). Based on the result, interaction networks between the 22 metabolites and 119 transcripts were organized in Hongyoung and Jayoung (Fig. 3 and Supplementary Table S6). The networks showed that the 119 transcripts were grouped into five clusters (I–V) (Table 2) and the 22 metabolites were divided into four groups (A–D) (Table 3). Metabolites in group A containing derivatives of a Pg, two Kas, a Dk, and an Ap were more predominant in Hongyoung compared with Jayoung, and were highly correlated with the transcripts in clusters I and II. On the other hand, group D metabolites including three Pt derivatives and one Dp derivative were more predominant in Jayoung and were showed to be highly connected with the transcripts in clusters IV and V. Group B and C metabolites were highly increased in both Hongyoung and Jayoung compared with Atlantic, although the increase of group C metabolites containing a Pg, three Pt, and an Mv derivative were a little more prominent in Jayoung than Hongyoung. The metabolites in groups B and C were shown to be strongly correlated with the transcripts in cluster III (Table 3 and Fig. 3).

**Table 2.** Classification of regulatory genes correlated with anthocyanin-related metabolites

Clusters^*a*^	Potato gene	Log_2_(fold change)		BLASTX TAIR10 Best Hit			BIN^*b*^
		Hongyoung	Jayoung	AGI	Annotation	Symbol	
Cluster I	PGSC0003DMT400049192	2.58	0.82	AT5G05600	2-Oxoglutarate (2OG) and Fe(II)- dependent oxygenase superfamily protein, leucoanthocyanidin dioxygenase	LDOX	BIN 16
	PGSC0003DMT400069897	2.96	0.98	AT2G39980	HXXXD-type acyl-transferase family protein	AT	BIN 16
	PGSC0003DMT400008366	3.07	1.24	AT4G00880	SAUR-like auxin-responsive protein family	SAUR31	BIN 17
	PGSC0003DMT400023715	–2.15	–0.55	AT1G56150	SAUR-like auxin-responsive protein family	SAUR71	BIN 17
	PGSC0003DMT400028801	2.37	0.77	AT2G37980	*O*-Fucosyltransferase family protein	OFT	BIN 17
	PGSC0003DMT400036081	2.64	0.95	AT1G05010	Ethylene-forming enzyme, 1-aminocyclopropane-1-carboxylate oxidase 4	ACO4	BIN 17
	PGSC0003DMT400040310	3.65	1.1	AT1G51760	Peptidase M20/M25/M40 family protein, IAA- alanine resistant 3, jasmonic acid responsive 3, jasmonoyl-l-isoleucine hydrolase	JIH	BIN 17
	PGSC0003DMT400064343	–2.27	–0.6	AT5G54510	Auxin-responsive GH3 family protein, IAA- amino acid synthase	GH3.6	BIN 17
	PGSC0003DMT400074744	3.1	0.79	AT1G15550	Gibberellin 3-oxidase 1	GA3OX1	BIN 17
	PGSC0003DMT400095956	–5.52	–1.76	AT2G26170	Cytochrome P450, family 711, subfamily A, polypeptide 1	MAX1	BIN 17
	PGSC0003DMT400014179	–3.79	–1.69	AT2G43060	ILI1-binding bHLH 1	IBH1	BIN 27.3
	PGSC0003DMT400016580	2.61	0.63	AT5G13220	Jasmonate zim domain protein 10	JAZ10	BIN 27.3
	PGSC0003DMT400018274	2.27	0.91	AT5G51790	bHLH DNA-binding superfamily protein	bHLH	BIN 27.3
	PGSC0003DMT400022651	3.74	1.34	AT1G30135	Jasmonate zim domain protein 8	JAZ8	BIN 27.3
	PGSC0003DMT400035445	2.63	1.11	AT4G24020	NIN-like protein 7	NLP7	BIN 27.3
	PGSC0003DMT400041580	2.05	0.9	AT5G65590	Dof-type zinc finger DNA-binding family protein, STOMATAL CARPENTER 1	SCAP1	BIN 27.3
	PGSC0003DMT400056352	2.66	0.84	AT5G13080	WRKY DNA-binding protein 75	WRKY75	BIN 27.3
	PGSC0003DMT400063260	2.76	1.22	AT5G23090	NUCLEAR FACTOR Y subunit B13	NF-YB13	BIN 27.3
	PGSC0003DMT400064428	–3.15	–1.25	AT3G15510	NAC domain containing protein 2	NAC2	BIN 27.3
	PGSC0003DMT400068332	2.34	0.88	AT3G02150	PLASTID TRANSCRIPTION FACTOR 1	PTF1	BIN 27.3
	PGSC0003DMT400070825	–2.18	–0.87	AT1G21150	Mitochondrial transcription termination factor family protein	AT1G21150	BIN 27.3
	PGSC0003DMT400071861	2.07	0.64	AT5G51780	bHLH DNA-binding superfamily protein	bHLH	BIN 27.3
	PGSC0003DMT400008273	2.36	0.74	AT4G11530	CYSTEINE-RICH RECEPTOR-LIKE PROTEIN KINASE 34	CRK34	BIN 30
	PGSC0003DMT400014960	–3.17	–1.26	AT5G24270	CALCINEURIN B-LIKE PROTEIN 4, SALT OVERLY SENSITIVE 3	CBL4/SOS3	BIN 30
	PGSC0003DMT400023467	–2.02	–0.65	AT3G49260	IQ-DOMAIN 21	IQD21	BIN 30
	PGSC0003DMT400027429	–2.96	–0.94	AT3G51550	Malectin/receptor-like protein kinase family protein, FERONIA	FER	BIN 30
	PGSC0003DMT400029325	–2.32	–0.97	AT1G01110	IQ-DOMAIN 18	IQD18	BIN 30
	PGSC0003DMT400034271	4.72	1.56	AT3G22060	Receptor-like protein kinase-related family protein	RLK	BIN 30
	PGSC0003DMT400037209	2.3	0.56	AT5G52390	PAR1 protein kinase	AT5G52390	BIN 30
	PGSC0003DMT400046778	2.05	0.57	AT4G20140	Leucine-rich repeat transmembrane protein kinase	LRRPK	BIN 30
Cluster II	PGSC0003DMT400020466	2.57	1.25	AT2G29740	UDP glucose: flavonoid-3-*O*-glucosyltransferase	UFGT	BIN 16
	PGSC0003DMT400066505	3.02	1.64	AT5G67150	Hydroxycinnamoyl-CoA shikimate/quinate hydroxycinnamoyl transferase	HCT	BIN 16
	PGSC0003DMT400070444	–6.26	–4.13	AT5G24530	2-Oxoglutarate (2OG) and Fe(II)-dependent oxygenase superfamily protein, flavanone 3-dioxygenase	F3H	BIN 16
	PGSC0003DMT400001145	2.74	2.03	AT5G48930	Hydroxycinnamoyl-CoA shikimate/quinate hydroxycinnamoyl transferase	HCT	BIN 16
	PGSC0003DMT400030504	3.77	3.15	AT5G54010	UDP-glycosyltransferase superfamily protein	UFGT	BIN 16
	PGSC0003DMT400045239	–2.38	–2.02	AT5G54160	*O*-Methyltransferase 1, flavonol 3′-O-methyltrasferase/caffeate O-methyltransferase	OMT1	BIN 16
	PGSC0003DMT400070465	–2.14	–1.68	AT1G55290	2-Oxoglutarate (2OG) and Fe(II)-dependent oxygenase superfamily protein, Feruloyl CoA ortho-hydroxylase 2	F6′H2	BIN 16
	PGSC0003DMT400076179	2.83	2.63	AT5G13930	Chalcone and stilbene synthase family protein	CHS	BIN 16
	PGSC0003DMT400004046	4.03	2.67	AT1G75750	GAST1 protein homolog 1	GAST1	BIN 17
	PGSC0003DMT400026627	2.9	1.38	AT3G23240	Ethylene response factor 1	ERF1	BIN 17
	PGSC0003DMT400037826	2.54	1.79	AT3G23240	Ethylene response factor 1	ERF1	BIN 17
	PGSC0003DMT400086139	2.98	1.52	AT1G15550	Gibberellin 3-oxidase 1	GA3OX1	BIN 17
	PGSC0003DMT400002125	2.89	2.03	AT1G68840	Related to ABI3/VP1 2	RAV2	BIN 27.3
	PGSC0003DMT400010253	2.72	1.53	AT4G36930	bHLH DNA-binding superfamily protein, SPATULA	SPT	BIN 27.3
	PGSC0003DMT400035433	2.54	1.22	AT4G24020	NIN-like protein 7	NLP7	BIN 27.3
	PGSC0003DMT400035439	5.61	3.83	AT4G24020	NIN-like protein 7	NLP7	BIN 27.3
	PGSC0003DMT400042922	4.27	2.1	AT3G56380	Response regulator 17	RR17	BIN 27.3
	PGSC0003DMT400043852	2.19	1.33	AT3G18990	AP2/B3-like transcriptional factor family protein, reduced vernalization response 1	VRN1	BIN 27.3
	PGSC0003DMT400045182	2.88	1.63	AT5G16770	Myb domain protein 9	MYB9	BIN 27.3
	PGSC0003DMT400070196	2.23	1.19	AT2G41690	Heat-shock transcription factor B3	HSFB3	BIN 27.3
	PGSC0003DMT400081478	–10.88	–6.03	AT1G51200	A20/AN1-like zinc finger family protein	AT1G51200	BIN 27.3
	PGSC0003DMT400002991	3.21	2.41	AT1G30135	Jasmonate zim domain protein 8	JAZ8	BIN 27.3
	PGSC0003DMT400014383	2.44	1.8	AT1G68320	Myb domain protein 62	MYB62	BIN 27.3
	PGSC0003DMT400035440	4.76	3.94	AT1G64530	Plant regulator RWP-RK family protein, NIN-like protein 6	NLP6	BIN 27.3
	PGSC0003DMT400045834	2.32	1.69	AT2G18550	Homeobox protein 21	HB21	BIN 27.3
	PGSC0003DMT400071481	2.29	1.25	AT1G07650	Leucine-rich repeat transmembrane protein kinase	LRRPK	BIN 30
	PGSC0003DMT400044827	–2.26	–1.68	AT4G16360	5′-AMP-activated protein kinase beta-2 subunit protein	KINβ2	BIN 30
	PGSC0003DMT400053964	3.39	3.13	AT4G23160	Cysteine-rich RLK (RECEPTOR-like protein kinase) 8	CRK8	BIN 30
Cluster III	PGSC0003DMT400001124	2.08	2.03	AT5G07990	Cytochrome P450 superfamily protein, Flavonoid 3′,5′-hydroxylase	F3′5′H	BIN 16
	PGSC0003DMT400001125	2.08	2.08	AT5G07990	Cytochrome P450 superfamily protein, Flavonoid 3′,5;-hydroxylase	F3′5′H	BIN 16
	PGSC0003DMT400016512	2.22	2.94	AT1G67980	Caffeoyl-CoA 3-*O*-methyltransferase	CCOMT	BIN 16
	PGSC0003DMT400025446	2.54	3.52	AT4G36220	Ferulic acid 5-hydroxylase 1	F5H	BIN 16
	PGSC0003DMT400030428	2.17	2.26	AT5G05270	Chalcone-flavanone isomerase family protein	CHI	BIN 16
	PGSC0003DMT400045220	2.41	2.74	AT2G23910	NAD(P)-binding Rossmann-fold superfamily protein, cinnamoyl-CoA reductase	CCR	BIN 16
	PGSC0003DMT400076178	2.4	3.29	AT5G13930	Chalcone and stilbene synthase family protein	CHS	BIN 16
	PGSC0003DMT400034486	–2.96	–4.05	AT1G29500	SAUR-like auxin-responsive protein family	SAUR66	BIN 17
	PGSC0003DMT400010235	8.06	8.04	AT2G23380	SET domain-containing protein, SETDOMAIN 1	SET1	BIN 27.3
	PGSC0003DMT400028253	–1.46	–2.04	AT1G08620	Transcription factor jumonji (jmj) family protein / zinc finger (C5HC2 type) family protein	PKDM7D	BIN 27.3
	PGSC0003DMT400033569	2.81	2.8	AT4G09820	bHLH DNA-binding superfamily protein, TRANSPARENT TESTA 8	TT8	BIN 27.3
	PGSC0003DMT400059950	3.33	3.2	AT3G14880	Transcription factor	AT3G14880	BIN 27.3
	PGSC0003DMT400007965	1.73	2.05	AT1G04830	Ypt/Rab-GAP domain of gyp1p superfamily protein	AT1G04830	BIN 30
	PGSC0003DMT400018720	–2.11	–2.25	AT1G56120	Leucine-rich repeat transmembrane protein kinase	LRRPK	BIN 30
	PGSC0003DMT400044824	–2.36	–2.47	AT4G16360	5′-AMP-activated protein kinase β2 subunit protein	KINβ2	BIN 30
	PGSC0003DMT400062862	1.94	2.36	AT5G50120	Transducin/WD40 repeat-like superfamily protein	WD40	BIN 30
	PGSC0003DMT400073082	–1.69	–2.04	AT5G45760	Transducin/WD40 repeat-like superfamily protein	WD40	BIN 30
	PGSC0003DMT400073506	–2.57	–3.17	AT3G21630	Chitin elicitor receptor kinase 1	CERK1	BIN 30
	PGSC0003DMT400073513	–4.56	–5.76	AT3G21630	Chitin elicitor receptor kinase 1	CERK1	BIN 30
Cluster IV	PGSC0003DMT400018670	–1.4	–2.67	AT1G17020	Fe(II)/ascorbate oxidase, senescence-related gene 1	SRG1	BIN 16
	PGSC0003DMT400022254	1.61	3.66	AT5G13930	Chalcone and stilbene synthase family protein	CHS	BIN 16
	PGSC0003DMT400029916	1.73	3.99	AT5G54060	UDP-glucose:flavonoid 3-*O*-glucosyltransferase	UFGT	BIN 16
	PGSC0003DMT400049165	1.32	2.44	AT5G13930	Chalcone and stilbene synthase family protein	CHS	BIN 16
	PGSC0003DMT400062562	1.18	2.95	AT5G54010	UDP-glycosyltransferase superfamily protein	UFGT	BIN 16
	PGSC0003DMT400030430	2.17	3.27	AT5G05270	Chalcone-flavanone isomerase family protein	CHI	BIN 16
	PGSC0003DMT400058554	2.02	2.91	AT4G22880	Leucoanthocyanidin dioxygenase	LDOX	BIN 16
	PGSC0003DMT400023914	1.12	2.1	AT1G22690	Gibberellin-regulated family protein	GRP	BIN 17
	PGSC0003DMT400030493	–0.83	–2.09	AT5G25900	GA requiring 3	GA3	BIN 17
	PGSC0003DMT400075778	1.04	2.52	AT5G37490	ARM repeat superfamily protein	ARM	BIN 17
	PGSC0003DMT400048325	4.37	6.46	AT1G76690	12-Oxophytodienoate reductase 2	OPR2	BIN 17
	PGSC0003DMT400066182	1.32	2.05	AT5G59845	Gibberellin-regulated family protein	GRP	BIN 17
	PGSC0003DMT400000838	1.55	2.8	AT5G19650	Ovate family protein 8	OFP8	BIN 27.3
	PGSC0003DMT400007511	1.6	3.94	AT1G19210	Integrase-type DNA-binding superfamily protein, ERF/AP2 transcription factor family	ERF/AP2	BIN 27.3
	PGSC0003DMT400021046	–1.03	–2.21	AT1G07360	CCCH-type zinc fingerfamily protein, MOS4- ASSOCIATED COMPLEX SUBUNIT 5A	MAC5A	BIN 27.3
	PGSC0003DMT400032803	–0.82	–2	AT5G14540	Protein of unknown function (DUF1421)	AT5G14540	BIN 27.3
	PGSC0003DMT400078477	2.69	4.63	AT1G22640	Myb domain protein 3	MYB3	BIN 27.3
	PGSC0003DMT400003078	1.67	2.9	AT2G40080	EARLY FLOWERING 4	ELF4	BIN 30
	PGSC0003DMT400016494	2.74	4.51	AT3G22840	Chlorophyll A-B binding family protein, EARLY LIGHT-INDUCABLE PROTEIN	ELIP	BIN 30
	PGSC0003DMT400044079	–2.75	–4.99	AT5G67385	Phototropic-responsive NPH3 family protein	AT5G67385	BIN 30
	PGSC0003DMT400070439	–1.06	–2.06	AT2G27030	Calmodulin 5	CAM5	BIN 30
	PGSC0003DMT400070451	–1	–2.22	AT1G21240	Wall-associated kinase 3	WAK3	BIN 30
	PGSC0003DMT400036503	–2.67	–3.87	AT1G70250	Putative receptor serine/threonine kinase, protease inhibitor/seed storage/LTP family protein	AT1G70250	BIN 30
	PGSC0003DMT400060792	2.46	3.64	AT3G47570	Leucine-rich-repeat protein kinase family protein	LRRPK	BIN 30
Cluster V	PGSC0003DMT400055529	0.24	3.92	AT3G53260	Phenylalanine ammonia lyase 2	PAL2	BIN 16
	PGSC0003DMT400058309	0.82	2.5	AT1G59960	NAD(P)-linked oxidoreductase superfamily protein	AT1G59960	BIN 16
	PGSC0003DMT400016816	–0.34	–2.06	AT1G10810	NAD(P)-linked oxidoreductase superfamily protein	AT1G10810	BIN 17
	PGSC0003DMT400037644	–0.11	–5.11	AT2G14960	Auxin-responsive GH3 family protein	GH3.1	BIN 17
	PGSC0003DMT400073092	–0.01	–2.23	AT4G18710	Protein kinase superfamily protein, brassinosteroid-insensitive 2	BI2	BIN 17
	PGSC0003DMT400000578	–0.4	–2.39	AT1G02080	Transcription regulators	AT1G02080	BIN 27.3
	PGSC0003DMT400023322	0.62	2.68	AT2G47460	Myb domain protein 12	MYB12	BIN 27.3
	PGSC0003DMT400027378	–0.43	–2.25	AT1G29280	WRKY DNA-binding protein 65	WRKY65	BIN 27.3
	PGSC0003DMT400048133	–0.65	–2.34	AT3G11280	Duplicated homeodomain-like superfamily protein	AT3G11280	BIN 27.3
	PGSC0003DMT400056008	–0.56	–2.18	AT5G47430	DWNN domain, a CCHC-type zinc finger	AT5G47430	BIN 27.3
	PGSC0003DMT400058234	–0.2	–2.75	AT1G70150	Zinc ion binding	AT1G70150	BIN 27.3
	PGSC0003DMT400066370	–0.38	–2.53	AT4G17570	GATA transcription factor 26	GATA26	BIN 27.3
	PGSC0003DMT400075906	–0.29	–2.12	AT4G16110	Response regulator 2	RR2	BIN 27.3
	PGSC0003DMT400064403	0.34	2.36	AT4G27280	Calcium-binding EF-hand family protein	CBP	BIN 30
	PGSC0003DMT400079206	0.11	2.6	AT4G08950	Phosphate-responsive 1 family protein, EXORDIUM	EXO	BIN 30
	PGSC0003DMT400079207	0.96	3.97	AT4G08950	Phosphate-responsive 1 family protein, EXORDIUM	EXO	BIN 30
	PGSC0003DMT400079208	0.41	3.26	AT4G08950	Phosphate-responsive 1 family protein, EXORDIUM	EXO	BIN 30
	PGSC0003DMT400079209	0.4	2.11	AT4G08950	Phosphate-responsive 1 family protein, EXORDIUM	EXO	BIN 30

AGI, Arabidopsis Genome Initiative Number; TAIR, The Arabidopsis Information Resource.

^*a*^ Regulatory genes were clustered according to gene and metabolite correlation in Fig. 3, which was calculated using Pearson correlation coefficients (*R* ^2^).

^*b*^ BINs of genes generated according to MapMan classification using the MapCave tool (http://mapman.gabipd.org/web/guest/mapcave)

**Table 3.** Classification of anthocyanin metabolites correlated with anthocyanin-related genes

	Log_2_(fold) ratio of:		
	Hongyoung:Atlantic	Jayoung:Atlantic	Jayoung:Hongyoung
Group A			
Pelargonidin-Rham-2Hex	5.45	3.63	–1.82
Kaempferol-Rham-2Hex	5.57	3.05	–2.52
Dihydrokaempferol-Feruloyl-2Hex	6.68	4.06	–2.62
Apigenin-Feruloyl-Rham-Hex	4.63	2.42	–2.20
Kaempferol-Feruloyl-Rham-Hex	8.16	4.40	–3.76
Group B			
Apigenin-Rham-Hex	5.12	3.90	–1.23
Dihydrokaempferol-Hex	6.79	4.94	–1.85
Apigenin-Coumaroyl-Rham-2Hex	7.86	7.06	–0.80
Pelargonidin-Coumaroyl-Rham-2Hex	9.17	8.17	–0.99
Pelargonidin-Feruloyl-Rham-2Hex	8.42	6.98	–1.44
Kaempferol-Rham-Hex	6.35	4.77	–1.58
Apigenin-Coumaroyl-Rham-Hex	5.39	4.43	–0.96
Dihydrokaempferol	7.29	5.67	–1.62
Group C			
Pelargonidin-Caffeoyl-Rham-2Hex	4.16	4.87	0.71
Petunidin-Coumaroyl-Rham-2Hex	6.83	9.25	2.43
Peonidin-Coumaroyl-Rham-2Hex	6.55	7.84	1.29
Peonidin-Feruloyl-Rham-2Hex	4.28	5.78	1.50
Malvidin-Coumaroyl-Rham-2Hex	4.44	6.66	2.22
Group D			
Petunidin-Caffeoyl-Rham-2Hex	2.37	8.61	6.24
Petunidin-Feruloyl-Rham-2Hex	>1	>1	6.45
Peonidin-Caffeoyl-Rham-2Hex	2.90	9.30	6.39
Delphinidin-Coumaroyl-Rham-2Hex	1.36	3.26	1.90

Validation of differential gene expression was performed for 14 genes using quantitative PCR (qPCR) with gene-specific primers (see Supplementary Table S1 available at *JXB* online). Real-time qPCR analysis with RNA isolated from sprouts of Hongyoung, Jayoung, and Atlantic showed that genes in clusters I and II (*JAZ8*, *JAZ10*, *WRKY75*, *ERF1*, and *MYB9*) were highly expressed in Hongyoung, whereas *MYB3* and *UFGT* (cluster IV) were highly expressed in Jayoung. The expression levels of most genes in cluster III (*CCOMT*, two *CHI*, two *CHS*, and *LODX*) were similarly increased in both Hongyoung and Jayoung (Supplementary Fig. S6 available at *JXB* online).

### Transcripts responsible for the differential accumulation of each anthocyanin group

#### Anthocyanin biosynthesis

The distribution of anthocyanins in the colored potatoes showed that Pg-derivative anthocyanins were abundant in light-red-colored Hongyoung, whereas Pn, Dp, Pt, and Mv derivatives of anthocyanins were enriched in dark-purple Jayoung. Transcripts related to anthocyanin biosynthesis were differentially regulated in each cultivar. Among the 10 genes related to anthocyanin biosynthesis in clusters I and II, homologs for leucoanthocyanidin dioxygenase (*LDOX*), acyl-transferase, a UDP-glucose:flavonoid *O*-glycosyltransferase (*UFGT*: PGSC0003DMT400020466), and hydroxycinnamoyl-CoA shikimate/quinate hydroxycinnamoyl transferases (*HCT*: PGSC0003DMT400066505) were more strongly up-regulated in light-red Hongyoung compared with Jayoung. In contrast, two homologs from cluster V [phenylalanine ammonia-lyase (*PAL2*) and NAD(P)-linked oxidoreductase] and six homologs from cluster IV (two *UFGT*s, two chalcone synthases (*CHS*s), a chalcone isomerase (*CHI*) and an ascorbate oxidase) were more strongly up- or down-regulated in dark-purple Jayoung compared with Hongyoung.

#### Hormones

Of the 12 genes implicated in hormone response in cluster I and four in cluster II (Table 2 and Fig. 3), homologs of a SAUR-like auxin-responsive protein (*SAUR71*), an IAA-amido synthase (*GH3.6*), and more axillary branches 1 (*MAX1*) were significantly down-regulated in Hongyoung compared with Atlantic. However, nine homologs (five in cluster I and four in cluster II) were significantly up-regulated in Hongyoung (Table 2). Hormone-related transcripts of clusters IV and V were highly correlated with group C and D metabolites that were highly increased in Jayoung compared with Hongyoung. The significant down-regulation of four transcripts was observed in Jayoung, including homologs of GA requiring 3 (*GA3*), *GH3.1*, brassinosteroid-insensitive 2 (*BI2*), and an NAD(P)-linked oxidoreductase protein. The four transcripts including gibberellin-regulated family proteins (AT1G22690 and AT5G59845) were up-regulated.

#### Transcription factors

Transcription factors in clusters I and II were demonstrated to be strongly connected with group A metabolites abundant in Hongyoung. Of these, four transcripts including A20/AN1-like zinc finger family protein were down-regulated, and 21 were up-regulated including *NAC2*, jasmonate zim domain protein 8 (*JAZ8*), *JAZ10*, *WRKY75*, *MYB9*, and *MYB62* (Table 2 and Fig. 3). In cluster III, which was connected with both group B and C metabolites, a homolog of jumonji (jmj) family protein (*PKDM7D*) was significantly down-regulated and the genes encoding set domain 1 (SET1), TT8, and a transcription factor were up-regulated in both Hongyoung and Jayoung.

Moreover, 13 transcripts encoding transcription factors (five in cluster IV and eight in cluster V) were highly correlated with group C and D metabolites abundant in Jayoung (Table 2 and Fig. 4). Of the five transcripts in cluster IV, transcripts for MOS4-associated complex subunit 5A (*MAC5A*) and a transcription factor were down-regulated and three transcripts for ovate family protein 8 (*OFP8*), an ERF/AP2 transcription factor, and *MYB3* were significantly up-regulated in Jayoung compared with Atlantic. In cluster V, the up-regulation of *MYB12* and the down-regulation of seven transcripts including *WRKY65*, two zinc finger transcription factors, response regulator 2 (*RR2*), and GATA transcription factor 26 (*GATA26*) were observed in Jayoung.

#### Signaling

The expression levels of 30 transcripts encoding proteins implicated in signaling pathways were correlated with flavonoid levels in Hongyoung and/or Jayoung (Table 2 and Fig. 3). Six homologs (four in cluster I and two in cluster II) of genes were significantly up-regulated and five were down regulated both in Hongyoung and Jayoung. The up- or down-regulation of transcripts related to signaling in clusters I and II were more significant in Hongyoung than in Jayoung. Furthermore, the significant down-regulation of genes encoding a phototropic-responsive NPH3 family protein, calmodulin 5 (CAM5), wall-associated kinase 3 (WAK3), and a protease inhibitor/seed storage/LTP family protein in cluster IV were observed in Jayoung. Eight transcripts (three in cluster IV and five in cluster V) were significantly up-regulated, including homologous genes encoding early flowering 4 (ELF4), early light-inducible protein (ELIP), and a leucine-rich repeat protein kinase in cluster IV and a calcium-binding EF-hand family protein (CBP) and exordium (EXO) in cluster V (Table 2 and Fig. 3)

## Discussion

In this study, as an effort to elucidate the differential regulation of anthocyanin biosynthesis involved in differential pigmentation of potatoes, a correlation test was performed in light-red-colored Hongyoung and dark-purple Jayoung with 22 anthocyanins and 167 genes categorized to flavonoid metabolism, hormones, transcription factors, and signaling. Of the 167 genes, 119 genes were strongly correlated with the 22 anthocyanins, and the correlation network showed that the genes and metabolites were divided into five clusters (I–V) and four subgroups (A–D) (Fig. 3, Tables 2 and 3). Many of the differentially expressed genes between white and colored potatoes coincided with a recent report performed with potato cultivars “Xin Dang” (white skin and white flesh) and “Hei Meiren” (purple skin and purple flesh) (Liu *et al.*, 2015). In particular, a CHI gene (PGSC0003DMT400030430), two CHSs (PGSC0003DMT400049165 and PGSC0003 DMT400076178), an LDOX (PGSC0003DMT400058554), a flavonoid 3′,5′-hydroxylase (F3′5′H; PGSC0003DMT 400001124), and a basic helix–loop–helix (bHLH) DNA-binding superfamily protein, transparent testa 8 (TT8: PGSC0003DMT400033569) that are increased in the skin and flesh of the purple potato “Hei Meiren” (Liu *et al.*, 2015) were also found to be increased in Hongyoung and Jayoung. However, the relative expression levels of these genes were differential between Hongyoung and Jayoung. Each gene cluster might be functionally connected to the anthocyanin subgroups, regulating the biosynthesis of anthocyanin derivatives that determined the colors of potatoes. Genes in cluster III were shown to be strongly connected with anthocyanins in groups B and C, which were highly accumulated in both light-red Hongyoung and dark-purple Jayoung. The genes in cluster III contained CHS, CHI, and F3′5′H. Expression of these genes was similar in Hongyoung and Jayoung; thus, these genes might be commonly involved in the biosynthesis of anthocyanins in both Hongyoung and Jayoung. Furthermore, genes encoding TT8 and WD40-repeat protein (WD40) in cluster III were strongly up-regulated in both Hongyoung and Jayoung. TT8, a bHLH-type regulation factor, forms a ternary complex with WD40-repeat protein and R2R3–MYB (WD40/bHLH/R2R3–MYB complex); these proteins are involved in the regulation of flavonoid pathways, and specifically in anthocyanin and pro-anthocyanin biosynthesis in Arabidopsis, purple cauliflower, and purple strawberry (Chiu and Li, 2012; Jaakola, 2013; Schaart *et al.*, 2013; Xu *et al.*, 2013). Studies on *TT8* promoter activity using WD40 (*ttg1*), bHLH (*tt8*, *gl3*, and *egl3*), and R2R3–MYB (*tt2*, *myb5*, *pap1*, and *pap2*) in Arabidopsis mutants showed that the *TT8* promoter activity is differentially regulated by various WD40/bHLH/R2R3–MYB complexes (Chiu and Li, 2012; Xu *et al.*, 2013). In our study, the up-regulation of homologous genes encoding TT8 and WD40 in both Hongyoung and Jayoung indicated that the accumulation of anthocyanin in red-light- and dark-purple-colored potato cultivars was commonly regulated by the TT8-mediated pathway.

Of the genes in clusters I and II, we observed that jasmonic acid (JA) signaling-related genes, including *JIH*, *JAZ8*, and *JAZ10*, were up-regulated in light-red Hongyoung compared with dark-purple Jayoung. JA has been known to increase anthocyanin production and to stimulate gene expression of CHS and UFGT. In Arabidopsis, JA activates the degradation of JAZs, a negative regulator of JA, in a SCF^COl1^ complex-dependent manner, to abolish the interaction between JAZs and the bHLH/R2R3–MYB complexes, and to stimulate activation of the WD40/bHLH (GL3, EGL3, and TT8)/R2R3–MYB (GL1 and MYB75) complexes, thereby activating the expression of anthocyanin biosynthesis-related genes (Qi et al., 2011, 2013). Of JA and its oxylipin derivatives, JA–Ile (but not JA, methyl jasmonate, or 12-oxo phytodienoic acid) promotes degradation of JAZ by the formation of the SCF^COl1^–JAZ complexes (Thines *et al.*, 2007). JIH catalyzes the cleavage of the JA–Ile conjugate, generating 2-hydroxy-JA. NaJIH-suppressed transgenic tobacco plants showed a dramatic increase in JA–Ile levels during herbivore attacks, thereby enhancing their resistance compared with that of wild-type tobacco (Woldemariam *et al.*, 2012). Thus, the up-expression of JIH most likely reduces JA–Ile levels. Taken together, the transcriptional up-regulation of homologous genes encoding JIH, JAZ8, and JAZ10 probably indicates that JAZ degradation-mediated anthocyanin biosynthesis might be inactivated in light-red Hongyoung.

In this study, most auxin-related genes were shown to be negatively correlated with anthocyanin levels. Genes encoding SAUR66, SAUR71, GH3.6, and MAX1 in clusters I and II were negatively correlated with group A or B anthocyanins that are abundant in light-red Hongyoung. Moreover, we observed a negative correlation between the levels of GH3.1 in cluster V and anthocyanins in group D that are enriched in dark-red Jayoung. *Auxin-sensitive SAUR66* and *SAUR71* genes are responsive to *GH3.1* and *GH3.6* genes that catalyze the conjugation of amino acids to auxin. MAX1, a member of the CYP711A cytochrome P450 family, has been known to down-regulate genes involved in the flavonoid pathway, including CHS, CHI, F3H, F3′H, FLS, DFR, ANS, and UFGT (Lazar and Goodman, 2006). Liu *et al.* (2014) showed that auxins regulated the expression levels of anthocyanin biosynthesis genes in red pap1-D Arabidopsis cells, including genes for six transcriptional factors (*TTG1*, *EGL3*, *MYBL2*, *TT8*, *GL3*, and *PAP1*) and four structural genes (*PAL1*, *CHS*, *DFR*, and *ANS*) (Liu *et al.*, 2014). The results demonstrated the involvement of auxin in anthocyanin biosynthesis. In other words, the anthocyanin biosynthesis in light-red Hongyoung and dark-purple Jayoung might be regulated by the inactivation of negative regulators, including MAX1.

In contrast to the down-regulation of auxin-related genes, genes involved in ethylene were up-regulated in the two pigmented potatoes compared with the white Atlantic, including homologous genes encoding 1-aminocyclopropane-1-carboxylate oxidase 4 (ACO4) in cluster I, an ethylene response factor 1 (ERF1) in cluster II, and an ERF/AP2 transcription factor in cluster IV. ACO4 converts 1-aminocyclopropane-1-carboxylic acid to ethylene, and ERF1 promotes ethylene production via the ethylene signaling cascade. The up-regulation of ACO4 and ERF1 in both Hongyoung and Jayoung indicated that ethylene may be related to the pigmentation of the two pigmented potatoes. In a recent study, exogenous treatment with the ethylene-releasing compound 2-chloroehtylphosphonic acid was reported to result in the accumulation of anthocyanins in grape skins and to stimulate the long-term expression of CHS, F3H, ANS, and UFGT (El‐Kereamy *et al.*, 2003). These results indicated that ethylene was involved in anthocyanin biosynthesis. However, a gene in cluster I encoding NAC2 (also called ANAC092 and ORE1), a positive regulator of ethylene-mediated leaf senescence, was observed to be down-regulated in both Hongyoung and Jayoung compared with Atlantic. Transcriptional expression of NAC2 has been shown to be up-regulated by ethylene insensitive 2 (EIN2), which activates ethylene signaling and induces the expression of senescence-associated genes (Woo *et al.*, 2013). Therefore, the result indicates that anthocyanin biosynthesis in the two pigmented potatoes may not be induced in a senescence-dependent manner activated by the EIN2–NAC2 pathway. Indeed, it has been reported that ethylene suppresses sugar-induced anthocyanin accumulation in Arabidopsis by suppressing the expression of positive regulators of the WD40/bHLH/R2R3–MYB complex and stimulating the expression of the negative R3–MYB regulator MYBL2 (Jeong *et al.*, 2010). Thus, ethylene differentially regulates anthocyanin biosynthesis according to developmental and environmental stimuli. However, studies into their regulation mechanisms are lacking.

In addition to the above genes, Fig. 3 showed that a large number of genes were connected with diverse anthocyanins. Quantitative changes in the secondary metabolites in groups A and B, which were most abundant in the light-red Hongyoung, showed negative or positive correlations with transcriptional changes in genes in clusters I and II. Moreover, the anthocyanin contents in groups C and D, which were most abundant in the dark-purple Jayoung, were negatively or positively correlated with the expression levels of genes in clusters IV and V (Table 2 and Fig. 3). In contrast, the expression level of two F3′5′Hs was not significantly different between Hongyoung and Jayoung (Table 2), while the Dp and Pt derivatives of anthocyanin were highly increased in Jayoung (Table 3). As the biosynthesis of blue Dp-type anthocyanins are known to be driven by the activity of F3′5′H (Ishiguro *et al.*, 2012), there might be other genes responsible for the Dp-based anthocyanin biosynthesis in Jayoung. The more significant up-regulation of *LODX*, *UFGT*, *CHS*, and *CHI* in cluster IV and *PAL2* and NAD(P)-linked oxidoreductase (AT1G59960) in cluster V might have role in the more significant accumulation of Dp and Pt derivatives. LODX converts the colorless leucoanthocyanidins into the colored anthocyanidins, which are inherently unstable under physiological conditions (Lo Piero, 2015). The addition of a glucose moiety in the 3-OH positions of anthocyanidins by UFGT increases the hydrophilicity and stability of anthocyanidins, conveying the flux of flavonoid intermediates towards the synthesis of anthocyanins (Lo Piero, 2015). Putting these together, with the significant increase of early-step genes including *PAL2*, *CHS*, and *CHI* in clusters IV and V, it can be postulated that that UFGT and LODX might have role in driving the flux and accumulation of Dp-type anthocyanins in Jayoung.

In conclusion, we explored the regulatory network connected to anthocyanin biosynthesis using integrated analysis of the metabolome and transcriptome in sprouts of three different colored potatoes: light-red Hongyoung, dark-purple Jayoung, and white Atlantic. Correlation analysis between metabolites and regulatory genes identified the regulatory genes associated with anthocyanin metabolites and provided new insight into the regulatory mechanism underlying the biosynthesis of anthocyanin accumulation in colored potatoes. Moreover, a connection network between changes in transcriptional expression and metabolite levels according to the pigmentation was obtained. The dataset could be harnessed by researchers to utilize genetic approaches to clarify the mechanism of anthocyanin regulation.

## Supplementary Data

Supplementary data are available at *JXB* online.

**Table S1.** Primer list used in qPCR analysis.

**Table S2.** Exact mass of aglycones, sugars, and acylated groups found in anthocyanins and flavonoid glycosides.

**Table S3.** Identification of anthocyanin biosynthesis-related compounds with MS/MS spectra obtained in ESI^+^ and ESI^−^ modes.

**Table S4.** List of 167 genes categorized to hormones, signaling, transcriptional regulation, and flavonoid metabolism.

**Table S5.** The correlation matrix of metabolites (anthocyanins) and gene expression levels.

**Table S6.** Interaction value between 22 metabolites and 119 genes that has a strong correlation coefficient (*R* ^2^>0.9).

**Fig. S1.** Tubers and sprouts of *Solanum tuberosum* cvs Atlantic, Hongyoung, and Jayoung.

**Fig. S2.** Structure and molecular formulae of anthocyanidins.

**Fig. S3.** Score plots and S-plots of orthogonal partial least-squares discriminant analysis (OPLS-DA) in positive (A) and negative (B) modes.

**Fig. S4.** Identification of anthocyanin derivatives using MS^2^ fragmentation.

**Fig. S5.** The number of differently expressed genes among Hongyoung, Atlantic, and Jayoung.

**Fig. S6.** Quantitative real-time RT-PCR (qPCR) analysis of genes involved in anthocyanin biosynthetic pathway and putative transcriptional regulators according to different color potato cultivars.

Supplementary Data
