# Supplementary material for: Network analysis of the metabolome and transcriptome reveals novel regulation of potato pigmentation
Source: J Exp Bot. 2016 Jan 4;67(5):1519–33. doi: 10.1093/jxb/erv549 (PMC4762390; doi:10.1093/jxb/erv549)
Supplement: Supplementary Data [file supp_67_5_1519__index.html]

Network analysis of the metabolome and transcriptome reveals novel regulation of potato pigmentation — Network analysis of the metabolome and transcriptome reveals novel regulation of potato pigmentation — Supplementary Data 

# Network analysis of the metabolome and transcriptome reveals novel regulation of potato pigmentation

## Supplementary Data

Data files

- supplementary\_figures\_S1\_S6\_tables\_S1\_S3.pdf - Supplementary Data
- supplementary\_tables\_S4\_S6.xlsx - Supplementary Data
