# Supplementary material for: Network analysis of the metabolome and transcriptome reveals novel regulation of potato pigmentation
Source: J Exp Bot. 2016 Jan 4;67(5):1519–33. doi: 10.1093/jxb/erv549 (PMC4762390; doi:10.1093/jxb/erv549)
Supplement: Supplementary Data [file supp_erv549_supplementary_figures_S1_S6_tables_S1_S3.pdf]

Supplementary Fig. S1 (Cho et al 2015)

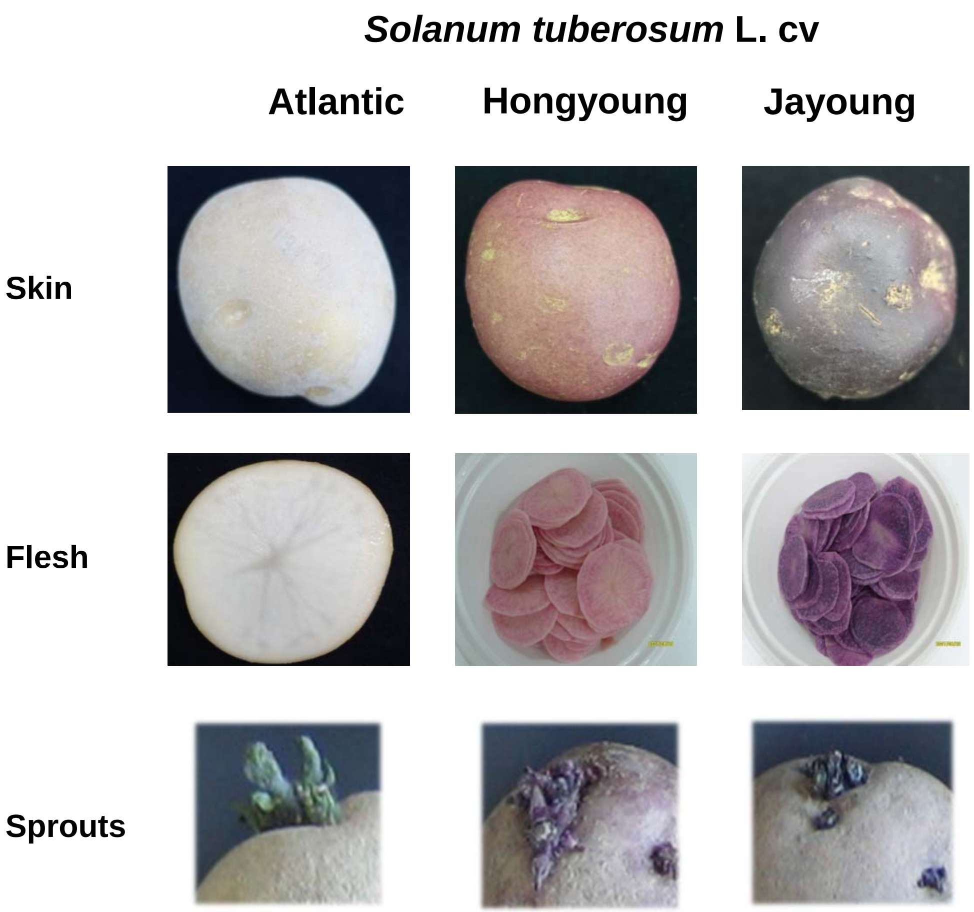

Fig. S1. Tubers and sprouts of *Solanum tuberosum* cv Atlantic, Hongyoung and Jayoung. Atlantic has white skin and white flesh, Hongyoung has light red skin, light-red flesh and light red sprouts, and Jayoung has dark purple skin, dark purple flesh and dark purple sprouts.

Supplementary Fig. S2 (Cho et al 2015)

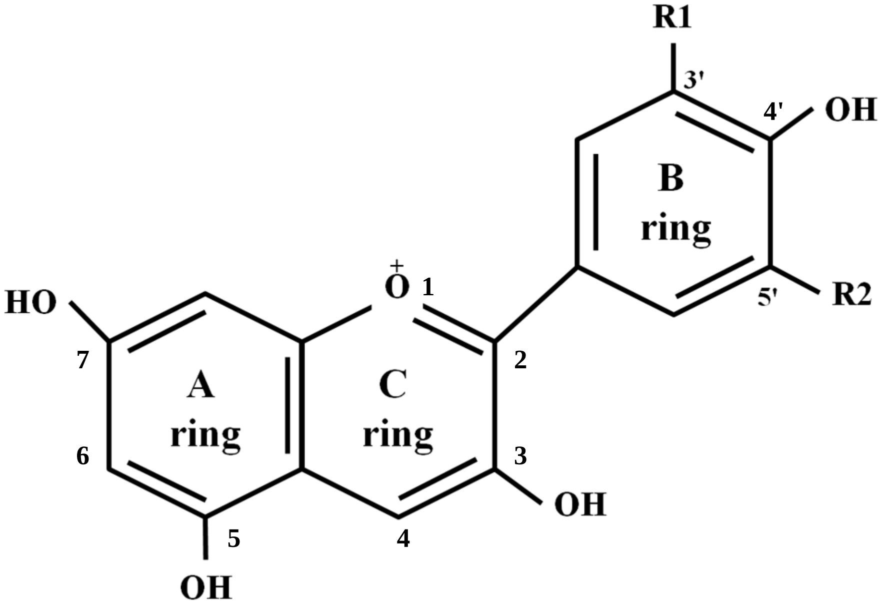

Anthocyanidin	R1	R2	Aglycone Formula
Cyanidin (Cy)	H	OH	$C_{15}H_{11}O_6^+$
Delphinidin (Dp)	OH	OH	$C_{15}H_{11}O_7^+$
Pelargonidin (Pg)	H	H	$C_{15}H_{11}O_5^+$
Peonidin (Pn)	OCH_3	H	$C_{16}H_{13}O_6^+$
Petunidin (Pt)	OCH_3	OH	$C_{16}H_{13}O_7^+$
Malvidin (Mv)	OCH_3	OCH_3	$C_{17}H_{15}O_7^+$

Fig. S2. Structure and molecular formula of anthocyanidins.
(Ref : Wu and Prior (2015) J. Agri.Food Chem 53,3101-3113)

Supplementary Fig. S3 (Cho et al 2015)

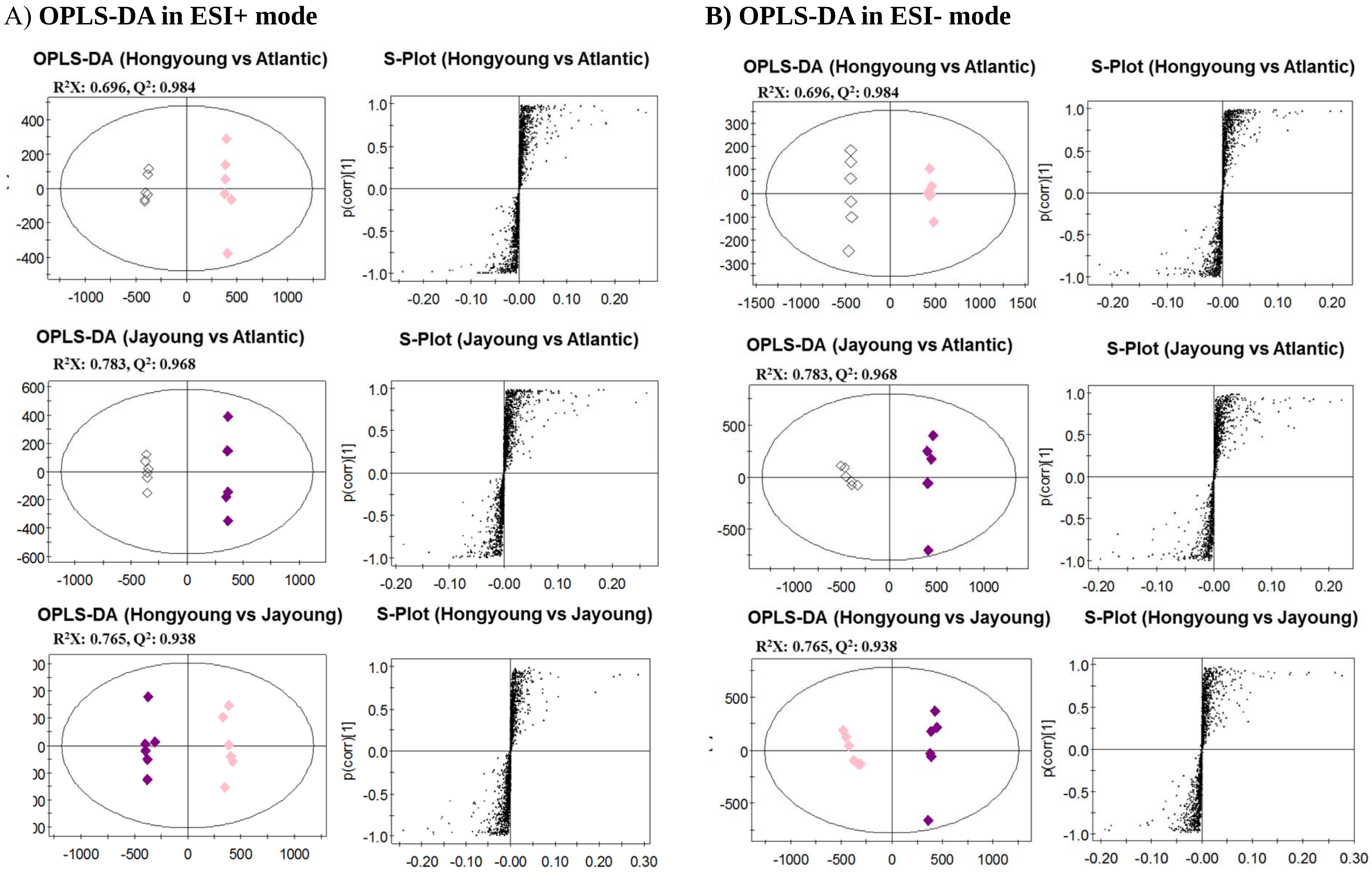

Fig. S3. Score plots and S plots of orthogonal partial least-squares discriminant analysis (OPLS-DA) in positive (A) and negative (B) modes. To evaluate metabolite mass ions that cause discrimination between Hongyoung and Atlantic, between Jayoung and Atlantic, and between Jayoung and Hongyoung, the *p*(corr) value of each mass ion were acquired from S plots of OPLS-DA. Open diamond : Athlantic, pink diamond : Hongyoung, purple diamond : Jayoun

Supplementary Fig. S4 (Cho et al 2015)

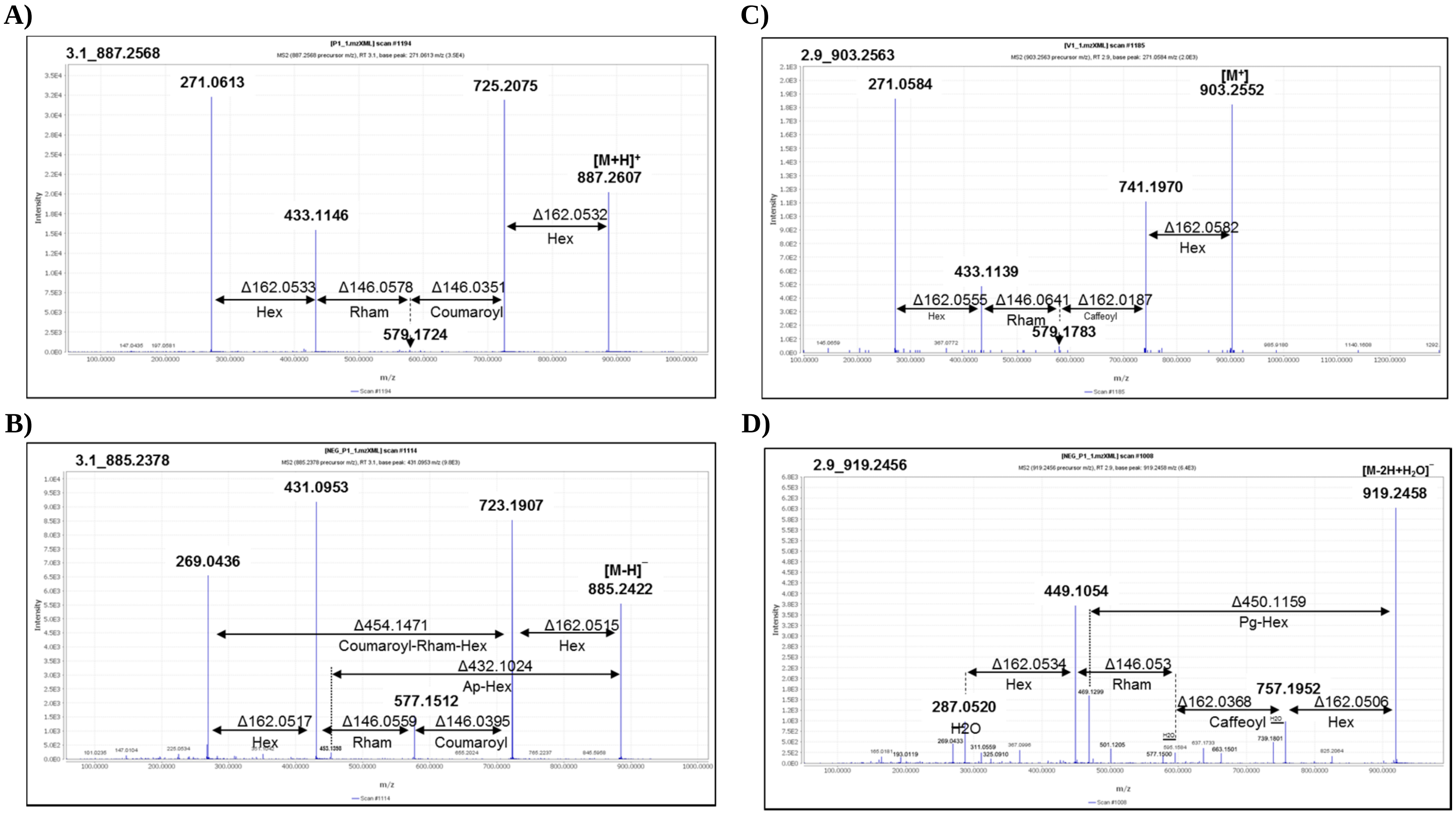

Fig. S4. Analysis of MS^2 fragmentation of flavonoids. MS^2 spectra of apigenin-coumaroyl-Rham-2 Hex in positive (A: 3.1_{RT}_887.2568) and in negative (B: 3.1_{RT}_885.2378) modes. MS^2 spectra of pelargonidin-caffeoyl-Rham-2 Hex in positive (C: 2.9_{RT}_903.2563) and in negative (D: 2.9_{RT}_919.2456) modes. Δ value represents neutral loss between two mass peaks and their identification is performed based on their exact mass values described in Supplementary Table 1. Compound abbreviated as follows: Hex, hexose; Rham, rhamnose; Caffeoyl, caffeic acid; Coumaroyl, coumaric acid; Ap, apigenin; Pg, pelargonidin.

Supplementary Fig. S5 (Cho et al 2015)

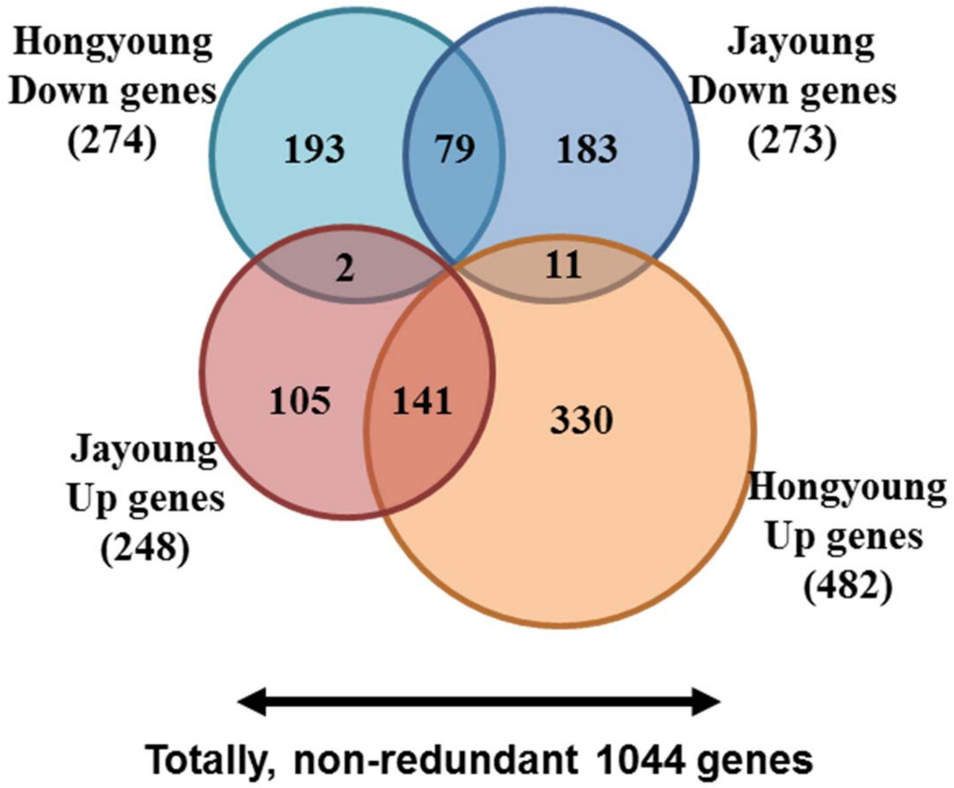

Fig. S5. The number of differently expressed genes comparing expression level of each gene between Hongyoung and Atlantic, between Jayoung and Atlantic and between Hongyoung and Jayoung.

Supplementary Fig. S6 (Cho et al 2015)

A)

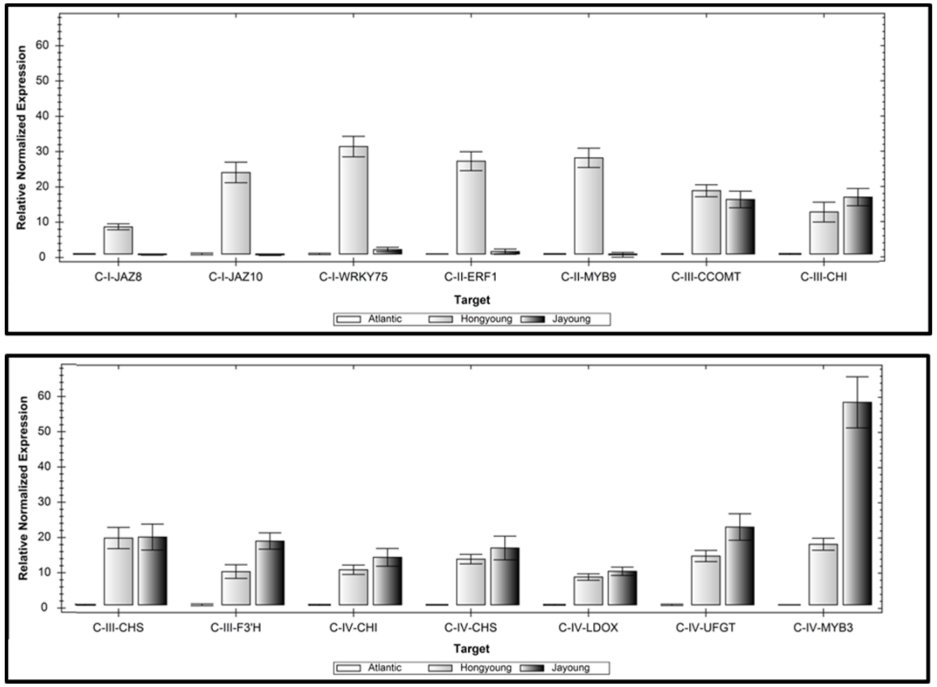

B)

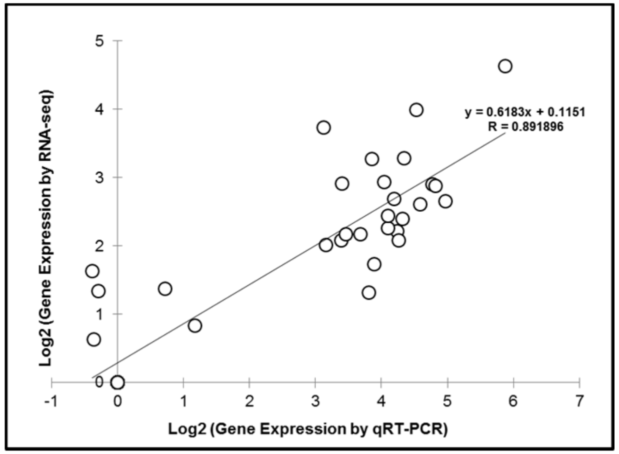

Fig. S6. Quantitative real-time RT-PCR (qPCR) analysis of genes involved in anthocyanin biosynthetic pathway and putative transcriptional regulators according to different color potato cultivars. A) transcriptomic qPCR analysis The expression level of each gene is normalized by using a reference gene, actin (X55752) and then calculated as relative level in a color potato to in Atlatic (control). qPCR data are presented as means ± SD for three biological replicates. *C-I-JAZ8* , PGSC0003DMT400022651 in Cluster I; *C-I-JAZ10*, PGSC0003DMT400016580 in Cluster I; *C-I-WRKY75*, PGSC0003DMT400056352 in Cluster I; *C-II-ERF1*, PGSC0003DMT400026627 in Cluster II; *C-II-MYB9*, PGSC0003DMT400045182 in Cluster II; *C-III-CCOMT*, PGSC0003DMT400016512 in Cluster III; *C-III-CHI*, PGSC0003DMT400030428 in Cluster III; *C-III-CHS*, PGSC0003DMT400076178 in Cluster III; *C-III-F3'H*, PGSC0003DMT400001125 in Cluster III; *C-IV-CHI*, PGSC0003DMT400030430 in Cluster IV; *C-IV-CHS*, PGSC0003DMT400049165 in Cluster IV; *C-IV-LDOX*, PGSC0003DMT400058554 in Cluster IV; *C-IV-UFGT*, PGSC0003DMT400062562 in Cluster IV; *C-IV-MYB3*, PGSC0003DMT400078477 in Cluster IV. The primer sequence for qPCR was described at supplemetary table S1. B) Correlation analysis between qPCR-based gene expression level and corresponding RNA-Seq data.

Table S1. Primer list used in qPCR analysis

	Transcript ID	Symbol	UniProt / TrEMBL	Gene annotation by The Potato Genome Sequencing Consortium (PGSC)	Name	Forward	Reverse
Cluster I	PGSC0003DMT400022651	JAZ8	M1AH03	Conserved gene of unknown function	C-I-JAZ8	GAGGAGGATAAAGAAAGCACAG	CAGTTTGAGGTTGTAATAATGGTG
Cluster I	PGSC0003DMT400016580	JAZ10	M1A7W1	Protein TIFY 9	C-I-JAZ10	ATATTTATCCAATAGCTCCGTCGT	ACTGTTGATTGCCTCCTTGTG
Cluster I	PGSC0003DMT400056352	WRKY75	Q8H9D7	WRKY-type DNA binding protein	C-I-WRKY75	TGATGATGGTTATAGATGGAGGA	TCGGTAGATTTGTCAATGGGA
Cluster II	PGSC0003DMT400026627	ERF1	M1ANG6	Ethylene response factor 10	C-II-ERF1	CGATGGCTATACTTAACTTTCCTG	TCTTGCTCTGTTTCTTCTACTCAC
Cluster II	PGSC0003DMT400045182	MYB9	M1BH83	R2R3-myb transcription factor	C-II-MYB9	ATATAAACAATCCACTTTCCACCA	TCTTTGATGAATTGTTGCTTAGTC
Cluster III	PGSC0003DMT400016512	CCOMT	M1A7S3	Caffeoyl-CoA O-methyltransferase	C-III-CCOMT	GCTTCTAAAGATCATGAATGCCA	AAATTGAATCTTATGCTCCACACC
Cluster III	PGSC0003DMT400030428	CHI	M1AU87	Chalcone isomerase	C-III-CHI	TGATGAAGAAATGGTACTTAGGTG	CCAAATACAATAGCCAACATGAC
Cluster III	PGSC0003DMT400076178	CHS	Q43163	Chalcone synthase 1B	C-III-CHS	CCAGCCCAAATCCAAGATAACTC	GTTGTTCTCAGCCAAGTCCT
Cluster III	PGSC0003DMT400001125	F3'H	M0ZHY8	Flavonoid 3',5'-hydroxylase	C-III-F3'H	CAAGGATGGGAATAGTGATGG	CCTCACATTACGATCTTTAGCA
Cluster IV	PGSC0003DMT400030430	CHI	M1AU88	Chalcone isomerase	C-IV-CHI	CTATTGTTAATGCTCCAGTTGAC	ACGATATCTTTGCACTCCCA
Cluster IV	PGSC0003DMT400049165	CHS	M1BNC5	Chalcone synthase 2	C-IV-CHS	ACTCTACTCCCTGATAGCGA	TAGAATTCCAATCAGAAATGCCT
Cluster IV	PGSC0003DMT400058554	LDOX	M1C2V1	Anthocyanin synthase	C-IV-LDOX	ATGAAGGGAAGTGGGTAACTG	GGTGGGAATTGAGATGGCTC
Cluster IV	PGSC0003DMT400062562	UFGT	M1C989	Anthocyanidin 3-O-glucosyltransferase	C-IV-UFGT	GCAGGATTCAGTTCAGTAATAGAG	CTTATCAACCTCCATCATCACC
Cluster IV	PGSC0003DMT400078477	MYB3	M1D070	Myb-like transcription factor 6	C-IV-MYB3	GAATTATGAGAAAGCCTTGTTGTG	CTATTAGTGACCACCTATTGCC
Reference	X55752	Actin		Actin	ACTIN	ATGGAACTGGAATGGTTAAGGCTGG	TGACCCATCCCTACCATAACACCG

Table S2. Exact mass of aglycones, sugars and acylated groups found anthocyanins and flavonoid glycosides.

compound	moleuclar fomula	exact mass	extact mass-H_2O
Flavanone			
naringenin	C15H12O5	272.07	254.06
Flavone			
apigenin	C15H10O5	270.05	252.04
luteolin	C15H10O6	286.05	268.04
tricetin	C15H10O7	302.04	284.03
Flavonol			
kaempferol	C15H10O6	286.05	268.04
quercetin	C15H10O7	302.04	284.03
myricetin	C15H10O8	318.04	300.03
Dihydroflavonol			
dihydrolkaempferol	C15H12O7	304.06	286.05
dihydrolquercetin	C15H12O8	320.05	302.04
dihydrolmyricetin	C15H12O9	336.05	318.04
Anthocyanidin			
pelargonidin (Pg)	C15H11O5+	271.06	
cyanidin (Cy)	C15H11O6+	287.06	
peonidin (Pn)	C16H13O6+	301.07	
petunidin (Pt)	C16H13O7+	317.07	
delphinidin (Dp)	C15H11O7+	303.05	
malvidin (Mv)	C17H15O7+	331.08	
Monosaccharides			
rhamnose (Rham)	C6H12O5	164.07	146.06
hexose (Hex)	C6H12O6	180.06	162.05
Acylated groups			
quinic acid	C7H12O6	192.06	174.05
coumaric acid (Coumaroyl)	C9H8O3	164.05	146.04
caffeic acid (Caffeoyl)	C9H8O4	180.04	162.03
ferulic acie (Feruloyl)	C10H10O4	194.06	176.05

Table S3. Identification of anthocyanin biosynthesis related compounds with MS/MS Spectra obtained in ESI+ and ESI− Modes

RT (min)	m/z		Tentative Identification	Formula	m/z		Fragment ions	
	ESI-	ESI+			ESI- (Δ ppm)	ESI+ (Δ ppm)	ESI-	ESI+
1.34	·	310.2118 [M+H]+	Dihydrocaffeoyl-spermidine	C16H27N3O3	·	310.2131 (4.1)	·	310.2123 (24), 293.1859 (32), 239.1410 (14), 222.1126 (100), 165.0536 (24), 123.0446 (25), 72.0805 (23)
1.83	147.0448 [M-H]⁻	149.0587 [M+H]+	Cinnamic acid	C9H8O2	147.0446 (1.3)	149.0603 (10.4)	·	·
1.84	164.0714 [M-H]⁻	166.0849 [M+H]+	Phenylalanine	C9H11NO2	164.0712 (1.5)	166.0868 (11.5)	·	166.0892 (2), 120.0816 (100), 103.0552 (80), 93.0706 (10), 91.0549 (20), 79.0544 (10), 77.0399 (50)
2.07	·	251.1386 [M+H]+	Caffeoyl-putrescine	C13H18N2O3	·	251.1396 (3.9)	·	251.1419 (5), 234.1137 (9), 163.0393 (100), 145.0267 (38), 135.0443 (33), 117.0344 (29), 89.0400 (24), 72.0816 (14)
2.18	353.0853 [M-H]⁻	·	Caffeoyl-quinic acid	C16H18O9	353.0873	·	353.0860 (13), 191.0561	·

					(5.6)		(100), 179.0342 (45), 135.0450 (44)	
2.18	757.2166 [M+H2O-2H]¯	741.2220 [M+]	Pelargonidin-Rham-2Hex	C33H41O19	757.2191 (3.3)	741.2242 (3)	757.2157 (65), 595.1591 (20), 501.1209 (30), 449.1057 (100), 431.1038 (10), 311.0534 (15), 287.0544 (45), 269.0432 (25), 193.0117 (20)	741.2231 (58), 579.1710 (34), 433.1135 (18), 271.0605 (100)
2.25	529.2991 [M-H]¯	531.3169 [M+H]+	Bis(dihydrocaffeoyl)-spermine	C28H42N4O6	529.3026 (6.6)	531.3183 (2.6)	529.3011 (100), 407.2636 (15), 365.2525 (20), 285.2280 (2.5), 243.2161 (2.5), 163.0397 (2.5), 121.0304 (2.5)	531.3163 (100), 367.2689 (3), 293.1868 (67), 222.1138 (82), 165.0547 (11), 123.0441 (5)
2.33	341.0859 [M-H]¯	*360.1285 [M+H+NH3]+	Caffeoyl-Hexose	C15H18O9	341.0873 (4.0)	·	341.0870 (4), 179.0335 (4), 135.0445 (100)	360.1285 (3), 181.0488 (67), 163.0373 (100), 135.0441 (80), 107.0515 (13)
2.45	·	181.0481[M+H]+	Caffeic acid	C9H8O4	·	181.0501 (11.0)	·	181.0490 (2), 163.0374 (9), 135.0442 (53), 107.0501 (100), 79.0550 (34), 77.0386 (77)
2.54	·	277.1653 [M+H]+	Coumaroyl-agmatine	C14H20N4O2	·	277.1665 (4.2)	·	277.1664 (17), 260.1391 (5), 235.1485 (3), 218.1194 (10), 147.0453 (100), 131.1297 (3), 119.0500 (21), 114.1034

								(7), 97.0766 (3), 91.0552 (7), 72.0830 (5)
2.57	577.1497 [M-H]⁻	579.1689 [M+H]+	Apigenin-Rham-Hex	C27H30O14	577.1557 (10.5)	579.1714 (4.3)	577.1539 (91), 269.0432 (100), 147.0067 (7)	579.1714 (23), 433.1110 (6), 271.0610 (100)
2.57	755.1977 [M-H]⁻	757.2159 [M+H]+	Kaempferol-Rham-2Hex	C33H40O20	755.2035 (7.7)	757.2191 (4.2)	755.1944 (49), 593.1472 (100), 285.0420 (38)	757.2150 (6), 611.1641 (18), 449.1097 (72), 287.0558 (100)
2.62	472.2415 [M-H]⁻	474.2593 [M+H]+	Bis(dihydrocaffeoyl)-spermidine	C25H35N3O6	472.2448 (6.9)	474.2604 (2.3)	472.2433 (100), 350.2052 (28), 308.1973 (42), 228.1699 (7), 186.1599 (7), 163.0384 (7), 121.0292 (7), 58.0321 (3)	474.2608 (77), 457.2359 (7), 310.2112 (3), 293.1873 (5), 236.1282 (15), 222.1140 (100), 165.0550 (21), 123.0444 (16), 100.0753 (3), 72.0817 (3)
2.67	·	307.1757 [M+H]+	Feruloyl-agmatine	C15H22N4O3	·	307.1770 (4.3)	·	307.1778 (37), 290.1519 (6), 261.0871 (7), 247.1453 (4), 177.0559 (100), 175.0875 (10), 158.0605 (34), 157.0763 (17), 149.0592 (7), 145.0282 (62), 133.0446 (9), 130.0633 (30), 117.0338 (13), 114.1011 (3), 89.0384 (4), 72.0823 (3)

2.72	470.2265 [M-H]¯	472.2435 [M+H]+	Caffeoyl-dihydrocaffeoyl-spermidine	C25H33N3O6	470.2291 (5.6)	472.2448 (2.7)	·	472.2446 (100), 310.2135 (17), 293.1847 (11), 234.1111 (11), 222.1117 (56), 165.0529 (8), 163.0372 (22)
2.75	675.1880 [2M-H]¯	·	Coumaroyl-quinic acid	C16H18O8	675.1925 (6.7)	·	675.1894 (2), 337.0923 (75), 191.0540 (100)	·
2.76	787.1989 [M-H]¯	·	Dihydrokaempferol-Feruloyl-2Hex	C37H40O19	787.2086 (12.3)	·	787.2081 (8), 449.1070 (100), 431.0991 (10), 337.0874 (17), 287.0620 (17), 269.0464 (20), 259.0630 (20), 191.0563 (13)	·
2.76	899.2186 [2M-H]¯	·	Dihydrokaempferol-Hex	C21H22O11	899.2246 (6.7)	·	899.2191 (13), 449.1100 (100), 287.0554 (17), 269.0465 (13), 259.0608 (8), 125.0245 (4)	·
2.77	449.1053 [M-H]¯	*451.1229 [M+H]+	Dihydrokaempferol-Hex	C21H22O11	449.1084 (6.9)	451.1240 (2.5)	449.1024 (15), 421.1104 (20), 287.0542 (65), 259.0597 (100)	451.0875 (10), 289.0723 (100), 271.0595 (31), 243.0647 (98), 215.0708 (67), 195.0282 (17), 153.0184 (80), 149.0236 (49), 107.0504 (18)
2.81	935.2400 [M+H2O-2H]¯	919.2500 [M+]	Delphinidin-Coumaroyl-Rham-2Hex	C42H47O23	935.2457 (6.1)	919.2508 (0.9)	935.2424 (100), 773.1845 (16), 755.1849 (18), 647.1624 (15), 621.1797 (10), 501.1252 (10), 481.0975 (4), 463.0818	919.2563 (100), 757.1982 (43), 465.1059 (28), 303.0516 (93)

							(5), 319.0425 (4), 301.0307 (7), 283.0236 (7), 193.0145 (5), 175.0037 (10)	
2.82	965.2502 [M+H2O-2H]⁻	949.2591 [M+]	Petunidin-Caffeoyl-Rham-2Hex	$C_{43}H_{49}O_{24}$	965.2563 (6.3)	949.2614 (2.4)	965.2528 (100), 825.2082 (6), 803.2017 (12), 785.1824 (6), 501.1184 (6), 495.1105 (19), 477.1037 (6), 333.0623 (8), 315.0493 (6), 297.0376 (3), 193.0162 (2)	949.2621 (100), 787.2059 (51), 479.1182 (25), 317.0660 (86)
2.90	367.1010 [M-H]⁻	369.1175 [M+H]+	Feruloyl-quinic acid	$C_{17}H_{20}O_9$	367.1029 (5.2)	369.1186 (2.9)	367.0999 (7), 191.0557 (100), 173.0439 (13), 134.0368 (14), 93.0354 (13)	369.1166 (1), 351.1049 (1), 195.0624 (1), 177.0550 (100), 149.0584 (6), 145.0274 (53), 134.0352 (3), 117.0331 (10), 89.0389 (5)
2.93	919.2434 [M+H2O-2H]⁻	903.2534 [M+]	Pelargonidin-Caffeoyl-Rham-2Hex (I)	$C_{42}H_{47}O_{22}$	919.2508 (8.1)	903.2559 (2.8)	919.2458 (100), 901.2360 (2), 825.2064 (3), 757.1952 (17), 739.1801 (8), 663.1501 (4), 637.1733 (6), 595.1584 (5), 577.1500 (4), 501.1205 (6), 469.1299 (25), 449.1054 (61), 367.0996 (6), 311.0559 (4), 287.0520 (18), 269.0433 (8)	903.2552 (95), 741.1970 (58), 433.1139 (25), 271.0584 (100)
2.95	949.2550 [M+H2O-2H]⁻	933.2652 [M+]	Peonidin-Caffeoyl-Rham-2Hex (I)	$C_{43}H_{49}O_{23}$	949.2614 (6.7)	933.2665 (1.4)	949.2590 (100), 931.2557 (5), 787.2055 (35), 769.1991 (12), 625.1704 (5), 479.1195 (76),	933.2654 (100), 771.2117 (46), 463.1248 (19),

							317.0648 (23), 299.0533 (12)	301.0702 (82)
2.96	919.245 [M+H2O-2H]⁻	903.2542 [M+]	Pelargonidin-Caffeoyl-Rham-2Hex (II)	C42H47O22	919.2508 (6.3)	903.2559 (1.9)	919.2515(100), 901.2311 (2), 825.2117 (4), 757.1962 (18), 739.1811 (7), 663.1547 (4), 637.1706 (5), 595.1627 (3), 577.1550 (3), 501.1242 (5), 469.1304 (19), 449.1059 (52), 367.1029 (8), 311.0550 (3), 287.0546 (18), 269.0434 (4)	903.2512 (100), 741.2010 (36), 433.1170 (17), 271.0585 (62)
3.01	949.2557 [M+H2O-2H]⁻	*933.2641 [M+]	Peonidin-Caffeoyl-Rham-2Hex (II)	C43H49O23	949.2614 (6)	933.2665 (2.5)	949.2587 (100), 931.2554 (4), 787.2053 (23), 769.1910 (14), 479.1195 (45), 317.0464 (18), 299.0581 (5)	933.2612 (100), 771.2119 (37), 463.1220 (18), 301.0705 (72)
3.04	693.3453 [M-H]⁻	695.3632 [M+H]+	Tri(dihydrocaffeoyl)-spermine	C37H50O9N4	693.3500 (6.7)	695.3656 (3.5)	693.3494 (100), 571.3110 (8), 529.2995 (14), 449.2742 (3), 407.2647 (6), 365.2534 (3), 285.2308 (2)	695.3613 (100), 531.3146 (5), 457.2335 (4), 367.2727 (1), 293.1877 (7), 222.1123 (15), 165.0535 (3), 123.0446 (1)
3.04	949.2558 [M+H2O-2H]⁻	*933.2643 [M+]	Petunidin-Coumaroyl-Rham-2Hex	C43H49O23	949.2614 (5.9)	933.2665 (2.3)	949.2583 (100), 809.2178 (3), 787.2089 (13), 769.1907 (5), 501.1256 (5), 495.1114 (10), 477.1046 (5), 333.0601 (5), 315.0471 (5), 297.0403 (5), 193.0123 (3)	933.2660 (100), 771.2123 (29), 479.1165 (17), 317.0670 (41)

3.07	979.2672 [M+H2O-2H]⁻	963.2773 [M+]	Petunidin-Feruloyl-Rham-2Hex	C44H51O24	979.2720 (4.9)	963.2770 (-0.3)	979.2683 (100), 817.2186 (28), 799.2017 (11), 691.1805 (4), 677.1688 (13), 651.1865 (4), 641.1685 (2), 501.1180 (7), 495.1102 (21), 477.1034 (6), 333.0593 (10), 315.0514 (11), 297.0396 (9), 193.0119 (3)	963.2778 (100), 801.2211 (38), 639.3135 (8), 493.3135 (17), 331.0844 (75)
3.08	885.2384 [M-H]⁻	887.2589 [M+H]+	Apigenin-Coumaroyl-Rham-2Hex	C42H46O21	885.2453 (7.8)	887.2610 (2.4)	885.2422 (59), 723.1907 (87), 577.1512 (18), 431.0953 (100), 269.0436 (68), 147.0104 (2)	887.2607 (61), 725.2075 (95), 433.1146 (47), 271.0613 (100)
3.08	903.2503 [M+H2O-2H]⁻	887.2589 [M+]	Pelargonidin-Coumaroyl-Rham-2Hex	C42H47O21	903.2559 (6.2)	887.2610 (2.4)	903.2523 (84), 741.2000 (22), 723.1873 (7), 647.1579 (11), 621.1752 (5), 595.1629 (3), 577.1546 (3), 501.1180 (8), 449.1059 (100), 311.0534 (7), 287.0544 (20), 269.0432 (8), 193.0098 (5)	887.2604 (50), 725.2071 (100), 433.1142 (42), 271.0609 (100)
3.13	933.2608 [M+H2O-2H]⁻	917.2692 [M+]	Peonidin-Coumaroyl-Rham-2Hex	C43H49O22	933.2665 (6.1)	917.2716 (2.6)	933.2639 (21), 771.2127 (22), 753.2012 (7), 479.1180 (100), 341.0650 (8), 317.0661 (20), 299.0546 (9)	917.2702 (71), 755.2172 (100), 463.1248 (48), 301.0727 (87)
3.14	163.0396 [M-H]⁻	·	Coumaric acid	C9H8O3	163.0395 (0.5)	·	·	·

3.14	979.2654 [M+FA+H2O-2H]⁻	917.2692 [M+]	Peonidin-Coumaroyl-Rham-2Hex	C43H49O22	979.2720 (6.7)	917.2716 (2.6)	979.2691 (22), 933.2682 (62), 771.2166 (24), 753.2012 (8), 479.1180 (100), 461.1096 (3), 317.0686 (19), 299.0570 (10)	917.2685 (76), 755.2161 (100), 463.1215 (26), 301.0704 (66)
3.17	1009.2753 [M+H2O-2H]⁻	947.2797 [M+]	Peonidin-Feruloyl-Rham-2Hex	C44H51O23	1009.2825 (7.2)	947.2821 (2.6)	1009.2775 (1), 963.2697 (78), 801.2173 (16), 783.2048 (11), 479.1168 (100), 341.0647 (6), 317.0659 (22), 299.0569 (10)	947.2784 (100), 785.2254 (36), 463.1242 (22), 301.0699 (65)
3.17	963.2708 [M+H2O-2H]⁻	947.2797 [M+]	Malvidin-Coumaroyl-Rham-2Hex	C44H51O23	963.2770 (6.5)	947.2821 (2.6)	963.27322 (40), 945.2715 (3), 801.2234 (17), 783.2106 (7), 509.1292 (100), 347.0762 (17), 329.0673 (10)	947.2806 (100), 785.2313 (88), 493.1369 (48), 331.0828 (86)
3.25	979.264 [M+H2O-2H]⁻	*917.2692 [M+]	Pelargonidin-Feruloyl-Rham-2Hex	C43H49O22	979.2720 (8.1)	917.2716 (2.6)	979.2510 (3), 933.2637 (100), 771.2094 (22), 753.2019 (5), 501.1166 (5), 449.1048 (90), 287.0542 (13), 269.0431 (9), 193.0119 (4)	917.2685 (76), 755.2161 (100), 433.1109 (21), 271.0611 (56)
3.26	593.1467 [M-H]⁻	595.1637 [M+H]+	Kaempferol-Rham-Hex	C27H30O15	593.1507 (6.7)	595.1663 (4.4)	593.1459 (78), 327.0488 (5), 285.0393 (100), 255.0293 (13), 227.0328 (6), 151.0049 (3)	595.1647 (2), 449.1125 (5), 287.0561 (100)
3.29	193.0496 [M-H]⁻	·	Ferulic acid	C10H10O4	193.0501 (2.5)	·	·	·
3.32	723.1884 [M-H]⁻	725.2052	Apigenin-Coumaroyl-	C36H36O16	723.1925	725.2082	723.1851 (100), 577.1534	725.2100 (26), 433.1138

		[M+H]+	Rham-Hex		(5.7)	(4.1)	(45), 269.0435 (95), 147.0090 (3)	(6), 271.0608 (100)
3.38	753.1958 [M-H]¯	755.2162 [M+H]+	Apigenin-Feruloyl-Rham-Hex	C37H38O17	753.2031 (9.7)	755.2187 (3.4)	753.2017 (100), 577.1493 (33), 269.0432 (60)	755.2208 (46), 433.1084 (6), 271.0613 (100)
3.47	636.2878 [M-H]¯	638.306 [M+H]+	Tri(dihydrocaffeoyl)-spermidine	C34H43O9N3	636.2921 (6.8)	638.3078 (2.8)	636.2916 (100), 514.2556 (6), 472.2432 (17), 392.2187(3), 350.2077 (6), 308.1972 (4), 228.1720 (2)	638.3069 (100), 474.2639 (43), 456.2517 (94), 310.2112 920, 293.1875 (62), 236.1281 (6), 222.1121 (79), 165.0553 (5), 123.0462 (2)
3.64	287.0535 [M-H]¯	·	Dihydrokaempferol	C15H12O6	287.0556 (7.2)	·	287.0564 (38), 269.0429 (7), 259.0619 (100), 243.0663 (23), 215.0713 (17), 201.0562 (20), 177.0539 (41), 173.0604 (16), 151.0031 (28), 125.0255 (95), 107.0136 (7), 83.0151 (13), 65.0051 (10), 63.0625 (8), 57.0375 (15)	·
4.05	769.1902 [M-H]¯	·	Kaempferol-Feruloyl-Rham-Hex	C37H38O18	769.198 (10.1)	·	769.4076 (100), 593.1574 (6), 285.0413 (6)	·
